# A Non‐Channel Function of CFTR: Attenuating Mitochondrial Oxidative Stress and Cardiomyocyte Senescence via Stabilization by USP45

**DOI:** 10.1111/acel.70610

**Published:** 2026-06-23

**Authors:** Chun Chen, Longtan Jiang, Yuewen Qiu, Chong Liu, Xiao Long, Pengcui Wu, Liang Li, Haixia Xu, Ping Deng, Li Yang, Qiao Jin

**Affiliations:** ^1^ Department of Cardiovascular Medicine, Hengyang Medical School The Changsha Central Affiliated Hospital, University of South China Changsha China; ^2^ Department of Cardiac Surgery, Xiangya Hospital Central South University Changsha China; ^3^ Department of Ultrasound Medicine, The Changsha Central Affiliated Hospital, Hengyang Medical School University of South China Changsha China; ^4^ Department of Medical Imaging, The Affiliated Changsha Central Hospital, Hengyang Medical School University of South China Changsha China; ^5^ Department of Radiology, The Affiliated Changsha Central Hospital, Hengyang Medical School University of South China Changsha China; ^6^ Department of Cardiovascular Medicine The Affiliated Zhuzhou Hospital Xiangya Medical College, Central South University Zhuzhou China; ^7^ Department of Cardiovascular Medicine The Third Xiangya Hospital (Central South University) Changsha China

**Keywords:** cardiomyocyte senescence, CFTR, mitochondrial oxidative stress, USP45

## Abstract

Cardiomyocyte senescence drives cardiovascular disease, underscoring the need to define its molecular mechanisms. The role of cystic fibrosis transmembrane conductance regulator (CFTR) ion channel in this process remains unclear, particularly regarding its expression and function. Atrial tissues were collected from patients with sinus rhythm or atrial fibrillation (AF) of varying durations. CFTR was downregulated in AF patients and negatively correlated with p16, p21, and p53. Myocardial aging models were established using D‐galactose (D‐gal) in both mice and neonatal mouse cardiomyocytes (CMs). In both animal and cellular models, D‐gal increased SA‐β‐gal positivity and senescence markers while decreasing CFTR. Overexpressing CFTR reduced D‐gal‐induced elevations in p16, p21, p53, and malondialdehyde (MDA), and restored superoxide dismutase (SOD), glutathione peroxidase (GSH‐Px), and catalase (CAT) activities. Mechanistically, CFTR alleviates mitochondrial oxidative stress damage by enhancing plasma membrane Ca^2+^ ATPase (PMCA) activity to reduce cytoplasmic Ca^2+^ levels. Furthermore, we identified USP45 as a direct binding partner of CFTR, which deubiquitinates CFTR by specifically targeting K48‐linked chains and the K688 residue. CFTR knockdown exacerbated D‐gal‐induced senescence and mitochondrial oxidative stress, which was rescued by USP45 overexpression. In conclusion, this study reveals a novel mechanism in which USP45‐mediated deubiquitination of CFTR mitigates cardiomyocyte senescence and mitochondrial oxidative stress, offering a targeted intervention against age‐related cardiovascular diseases.

## Graphical abstract

USP45 stabilizes CFTR through deubiquitination, thereby activating PMCA, which inhibits calcium/chloride overload and mitochondrial oxidative stress in senescent cardiomyocytes, ultimately alleviating cardiomyocyte senescence.

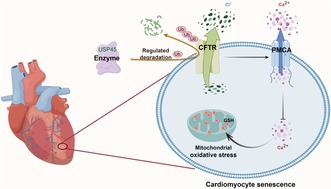

## 1 Introduction

Cardiomyocyte senescence is a critical risk factor for various cardiovascular diseases, including myocardial infarction, cardiac fibrosis, arrhythmia, heart failure, and atherosclerosis (Song et al. 2024). It can be triggered by multiple stressors such as DNA damage, oxidative stress, mitochondrial dysfunction, and epigenetic alterations (Zhang et al. 2024). Senescent cardiomyocytes exhibit impaired contractility, increased pacing frequency, pronounced mitochondrial dysfunction, and hypertrophic growth, collectively contributing to cardiac dysfunction and remodeling (Duan et al. 2023; Zhang et al. 2024). Recent studies have demonstrated that genetic or pharmacological elimination of senescent cells holds potential for alleviating certain features of cardiac aging (Maggiorani et al. 2024). Given the complexity of the mechanisms underlying cardiomyocyte senescence, understanding its key drivers and exploring targeted interventions are of substantial value for the development of novel therapeutic strategies for cardiovascular diseases.

The cystic fibrosis transmembrane conductance regulator (CFTR) is an adenosine triphosphate (ATP)‐gated anion channel that mediates the transport of Cl^−^ and HCO_3_^−^, playing a crucial role in fluid transport and homeostasis (Liu et al. 2024). Although CFTR is expressed in the heart, its cardiac functions remain incompletely understood (Wang et al. 2020). Emerging evidence indicates that CFTR is involved in cardiomyocyte electrophysiological activity, and its silencing leads to impaired contractile function (Akita et al. 2020; Sellers et al. 2010). CFTR deficiency has been associated with defective cardiac development in zebrafish embryos and is linked to dilated cardiomyopathy (Liu et al. 2020). Furthermore, dysregulated CFTR expression contributes to premature cellular senescence in murine lung tissue (Wellmerling et al. 2019), and its reduced expression in skeletal muscle impairs autophagy and myogenesis, thereby exacerbating skeletal muscle aging (Chen et al. 2025). However, the role of CFTR in cardiomyocyte senescence remains poorly investigated and warrants further exploration.

Mitochondria are involved in multiple interconnected cellular processes, including ATP production, nutrient metabolism, calcium homeostasis, and the regulation of programmed cell death (Ramaccini et al. 2020). Mitochondrial dysfunction is a hallmark of cardiomyocyte senescence. In aging cardiomyocytes, the balance of mitochondrial fission and fusion is disrupted, leading to compromised functional integrity (Tang et al. 2020). Accumulating evidence suggests that mitochondrial DNA mutations contribute to cardiovascular aging and related pathologies, while mitochondrial‐derived reactive oxygen species (ROS) may activate stress‐induced senescent phenotypes (Camacho‐Encina et al. 2024; Stefanatos and Sanz 2018). CFTR has been shown to confer protection against neuronal apoptosis following cerebral ischemia–reperfusion by mitigating mitochondrial oxidative stress (Zhang et al. 2018). Furthermore, CFTR‐deficient mouse models and human lung epithelial cells exhibit reduced glutathione (GSH) levels and elevated oxidative stress (Artusi et al. 2025). However, the role of CFTR in modulating mitochondrial oxidative stress in cardiomyocytes remains to be fully elucidated.

Ubiquitination plays a key role in regulating CFTR protein degradation and maintaining its peripheral quality control (Taniguchi et al. 2023). Knockdown of STUB1 was shown to upregulate CFTR expression and alleviate calcium oxalate crystal‐induced renal tissue injury in mice (Hou et al. 2024). In addition, ubiquitin protein ligase E3C (UBE3C) promotes the endoplasmic reticulum‐associated and peripheral degradation of misfolded CFTR (Kamada et al. 2023). Okiyoneda et al. (2018) proposed that the peripheral protein quality control system can effectively recognize and ubiquitinate partially unfolded CFTR, underscoring the involvement of ubiquitination in the maintenance of CFTR stability. Shaw et al. (2010) demonstrated that serum/glucocorticoid‐regulated kinase 1 (SGK1)‐mediated phosphorylation of neural precursor cell expressed developmentally down‐regulated 4–2 (Nedd4‐2) inhibits its ubiquitination of CFTR, thereby increasing CFTR membrane abundance—highlighting the regulatory role of ubiquitination in modulating CFTR levels. However, whether deubiquitinating enzymes modulate mitochondrial function and cellular senescence phenotypes in cardiomyocytes via CFTR remains unclear.

This study is designed to identify ubiquitination‐related enzymes that regulate CFTR protein stability through a series of in vivo and in vitro experiments, and to further elucidate the roles and underlying mechanisms by which these enzymes and CFTR modulate mitochondrial function and senescence‐associated phenotypes in cardiomyocytes. Atrial fibrillation (AF) is closely associated with cardiomyocyte senescence, and during disease progression, it promotes adverse atrial remodeling (Adili et al. 2022). The AF patient samples are used to indicate the clinical relevance of CFTR to myocardial senescence, and the subsequent mechanistic studies were conducted using an independent D‐galactose (D‐gal)‐induced senescence model, which does not involve direct investigation of arrhythmias. Our findings offer a theoretical foundation for developing novel therapeutic strategies against cardiovascular aging‐related diseases.

## 2 Methods and Materials

### 2.1 Clinical Data Collection

This study enrolled six patients with paroxysmal short‐term atrial fibrillation (SAF) and six with long‐standing permanent atrial fibrillation (LAF), all of whom underwent open‐heart surgery; six patients in sinus rhythm (SR) were included as controls. Patients older than 60 years of age (excluded to minimize age‐related confounding effects) or those with relevant comorbidities (e.g., malignant tumors or chronic inflammatory diseases) were not eligible for inclusion. Left atrial appendage (LAA) tissue samples were obtained from patients with AF and those in sinus rhythm during open‐heart surgery, rapidly frozen in liquid nitrogen, stored at −80°C, and subsequently used to analyze features of premature cellular aging in LAA tissue. All patients provided written informed consent and were admitted to Changsha Central Hospital. The study adhered to the principles outlined in the Declaration of Helsinki and was approved by the Ethics Committee of Changsha Central Hospital (2023‐151).

### 2.2 D‐Gal‐Induced Cardiac Aging Mouse Model

C57BL/6 male mice (6 months old, 20–30 g) were purchased from Hunan Silaike Jingda Laboratory Animal Company. The mice were housed under standard conditions: room temperature maintained at 22°C–25°C, relative humidity at 60% plus or minus 5%, and a 12‐h light/dark cycle. Food and water were freely available. After one week of acclimatization, mice were randomly divided into five groups (*n* = 5 per group): Sham group, D‐gal group, D‐gal+oe‐NC group, D‐gal+oe‐CFTR group, and D‐gal+oe‐USP45 group. Cardiac aging was induced by daily subcutaneous injection of D‐galactose (D‐gal, 120 mg/kg/day) into the posterior neck region for 12 weeks (Wang et al. 2023). One week before modeling, mice in the D‐gal+oe‐NC and D‐gal+oe‐USP45 groups received slow tail vein injections of lentiviral vectors (1 × 10^9^ viral particles/mouse) (Zhao et al. 2019). The USP45 overexpression lentiviral vector (oe‐USP45, Catalog number: HG‐LV027768) and the control vectors (oe‐NC) were purchased from Honorgene. After 12 weeks of modeling, transthoracic echocardiography was performed on mice under 3% isoflurane anesthesia using a Vevo 2100 high‐resolution imaging system (VisualSonics, FUJIFILM, Toronto, Canada) equipped with a 40.0 MHz phased‐array transducer. Cardiac function and structure were assessed based on changes in end‐systolic and end‐diastolic dimensions measured by M‐mode ultrasound. Ejection fraction (EF) and fractional shortening (FS) percentages, left ventricular anterior wall thickness at end‐systole (LVAW;s), left ventricular internal diameter at end‐systole (LVID;s), left ventricular posterior wall thickness at end‐systole (LVPW;s), left ventricular volume at end‐systole (LV Vol;s), left ventricular internal diameter at end‐diastole (LVID;d), left ventricular volume at end‐diastole (LV Vol;d), and left ventricular mass (LV Mass) were recorded. Following ultrasound examination, mice were euthanized by intraperitoneal injection of sodium pentobarbital (150 mg/kg). Cardiac tissues were collected for subsequent analysis. All animal experiments were conducted in compliance with the Guide for the Care and Use of Laboratory Animals and approved by the Animal Ethics Committee of the South China University Affiliated Changsha Central Hospital (No. 2022‐S0031).

### 2.3 Hematoxylin and Eosin (H&E) and Masson Staining

Cardiac paraffin sections (4 μm) were deparaffinized and rehydrated. For H&E staining, sections were stained with hematoxylin (AWI0001a, Abiowell; 5 min) and eosin Y (G1100, Solarbio; 1 min), dehydrated through graded ethanol (95%–100%, 5 min/step), cleared in xylene, and mounted. For collagen detection, Masson's trichrome staining was performed per kit instructions (AWI0253a, Abiowell). Histopathology was assessed by light microscopy.

### 2.4 Neonatal Mouse Cardiomyocytes (NMCMs) Extraction and Processing

Newborn mice were anesthetized by inhalation of 3% isoflurane and then euthanized via cervical dislocation. NMCMs were isolated according to the following procedure (Liang et al. 2015). Hearts were rapidly excised and transferred to a 35‐mm culture dish containing ice‐cold Hank's balanced salt solution (HBSS). The cardiac tissue was then minced into 1–3 mm^3^ fragments and placed in a centrifuge tube with 5 mL of 0.08% trypsin. The tube was incubated in a 37°C water bath under gentle agitation for 6 min. After allowing the fragments to settle for 3 min, the supernatant was carefully aspirated. Next, 5 mL of fresh 0.125% collagenase II was added, and the tube was agitated again at 37°C for 15 min. The supernatant containing released cells was gently transferred to a new 15 mL centrifuge tube containing 5 mL of complete DMEM medium (supplemented with 10% FBS and 1% penicillin–streptomycin) to terminate trypsin activity. This digestion cycle was repeated 2–5 times until most tissue fragments were dissociated. The pooled cell suspension was centrifuged at 1000 rpm for 5 min. The pellet was resuspended in 6 mL of complete medium and seeded into a large culture dish. After 2 h of incubation, the unattached cells (enriched in cardiomyocytes) were collected from the supernatant, centrifuged, and resuspended in complete DMEM medium containing BrdU. This cell suspension was then seeded into culture flasks and maintained at 37°C in a 5% CO_2_ incubator. Cells were passaged upon reaching confluence. The identity of NMCM was confirmed by immunofluorescence (IF) staining using an α‐actinin antibody.

NMCMs were treated with 40 μM D‐gal for 10 days to establish a cellular senescence model (Wang et al. 2023). In the D‐gal+TEMPO group, NMCMs were exposed to 0.1 mM TEMPO (2,2,6,6‐Tetramethylpiperidin‐1‐oxyl, HY‐W001187, MCE), a mitochondrial ROS‐specific antioxidant, for 2 h following the 48‐h D‐gal stimulation [26]. Plasmid transfection was performed 48 h prior to the induction with D‐gal. Prior to D‐gal induction, NMCMs were pretreated with the proteasome inhibitors MG132 (10 μM) (Varney et al. 2015) or carfilzomib (1 μM) (Halasi et al. 2014) for 12 h, forming the D‐gal+MG132 and D‐gal+carfilzomib groups, respectively.

### 2.5 Cells Transfection

All plasmids used in this study were purchased from Honorgene. These include short hairpin RNA (shRNA) constructs for knocking down mouse CFTR (sh‐CFTR#1/2/3; Catalog: HG‐SH021050) and the plasma membrane Ca^2+^ ATPase (PMCA) (sh‐PMCA#1/2/3; Catalog: HG‐SH1359507), overexpression plasmids for CFTR (oe‐CFTR; Catalog: HG‐MO021050) and USP45 (oe‐USP45; Catalog: HG‐MO027768), alongside their corresponding negative control vectors (sh‐NC and oe‐NC). Additionally, a series of ubiquitin mutant plasmids (K6, K11, K27, K29, K33, K48, K63; Catalogs: HG‐MO027768‐K6 to HG‐MO027768‐K63) and the K688R CFTR mutant plasmid (Catalog: HG‐MO021050‐K688) were also utilized. According to the manufacturer's protocol, plasmid transfection into NMCMs was performed using serum‐free medium and Lipofectamine 2000 (11668019, Invitrogen). After 6 h, the medium was replaced with fresh culture medium for continued incubation.

### 2.6 Senescence‐Associated β‐Galactosidase (SA‐β‐Gal) Staining

Mouse cardiac tissues were embedded in Optimal Cutting Temperature (OCT) compound and sectioned into 6‐μm‐thick frozen sections. Sections were immersed in distilled water to equilibrate to room temperature. SA‐β‐gal staining was performed using a β‐galactosidase staining kit (Cat# AWI0305a, Abiowell).

Briefly, sections were covered with 50 μL of SA‐β‐gal staining fixative solution and incubated at room temperature for 1 h. After washing with PBS, the staining working solution was applied to the sections. Sections were then incubated overnight at 37°C in a CO_2_‐free incubator. Following glycerol mounting, stained sections were examined and imaged under a light microscope (Model BA210T, Motic, China).

NMCMs were seeded in 6‐well plates. The cells were washed once with PBS and then fixed with 1 mL of β‐galactosidase staining fixative for 15 min. After three washes with PBS, 1 mL of staining working solution was added, and the plates were sealed with parafilm, followed by overnight incubation at 37°C. Staining results were observed under a microscope.

### 2.7 Western Blot (WB) Analysis

Mouse cardiac tissues or primary NMCMs were homogenized and lysed using RIPA lysis buffer. The protein concentration was determined with a BCA assay kit. Equal amounts of protein samples were separated by 10% SDS‐PAGE (75 V for 130 min) and subsequently transferred onto nitrocellulose (NC) membranes. The membranes were blocked with 5% skim milk for 90 min at room temperature, followed by incubation with specific primary antibodies overnight at 4°C. After washing, the membranes were incubated with the corresponding secondary antibodies for 90 min at room temperature. The protein bands were visualized using SuperECL Plus ultrasensitive luminescence solution (K‐12045‐D50, Advansta) and imaged with a ChemiScope 6100 imaging system (Clinx, China). Band intensity (gray value) was quantified using ImageJ software (National Institutes of Health, USA). GAPDH was used as the internal loading control. All antibody information is listed in Table 1.

**TABLE 1:** Antibody information used in WB.

Proteins name	Dilution ratio	Code	Molecular weight	Manufacturer
p16	1:2000	25,537–1‐AP	16–18 kDa	Proteintech
p21	1:2000	30,109–1‐AP	21 kDa	Proteintech
p53	1:10000	16,806–1‐AP	53 kDa	Proteintech
CFTR	1:1000	ab215203	150 kDa	Proteintech
PMCA	1:1000	66,511–1‐Ig	150 kDa	Abcam
USP45	1:500	12,053–1‐AP	70–100 kDa	ThermoFisher
USP10	1:500	24,559–1‐AP	100–130 kDa	Proteintech
USP33	1:1000	21,393–1‐AP	107 kDa	Abcam
USP9X	1:5000	66,514–1‐Ig	260–290 kDa	Proteintech
GAPDH	1:5000	68,460–1‐Ig	36 kDa	Proteintech
HRP goat anti‐mouse IgG	1:5000	SA00001‐1	/	Proteintech
HRP goat anti‐rabbit IgG	1:6000	SA00001‐2	/	Proteintech

### 2.8 Biochemical Kit Test

According to the manufacturers' instructions, the levels/activities of malondialdehyde (MDA), superoxide dismutase (SOD), glutathione peroxidase (GSH‐Px), and catalase (CAT) in mouse serum, cardiac tissue, and/or NMCMs, as well as the levels of adenosine triphosphate (ATP), the nicotinamide adenine dinucleotide (NAD^+^)/reduced nicotinamide adenine dinucleotide (NADH) ratio, calcium ions (Ca^2+^), and chloride ions (Cl^−^) within NMCMs were measured using commercial assay kits. Except for the NAD^+^/NADH assay kit (S0175, Beyotime, China), all other kits were purchased from Nanjing Jiancheng Bioengineering Institute (China), with the following catalog numbers: MDA (A003‐1‐2), SOD (A001‐3‐2), GSH‐Px (A005‐1‐2), CAT (A007‐1‐1), ATP (A095‐1‐1), Ca^2+^ (C004‐2‐1), and Cl^−^ (C003‐2‐1).

### 2.9 Enzyme‐Linked Immunosorbent Assay (ELISA)

Additionally, ELISA kits were used to measure the secretion levels of senescence‐associated secretory phenotype (SASP) factors, including interleukin‐6 (IL‐6), interleukin‐8 (IL‐8), tumor necrosis factor‐alpha (TNF‐α), and C‐C motif chemokine ligand 2 (CCL2), in NMCMs and mouse serum. Kits for IL‐6 (KE10007), TNF‐α (KE10002), and CCL2 (KE10123) were from Proteintech, and the IL‐8 (CXCL15) kit (EM1592) was from Fine Test (https://fntest.cn/).

### 2.10 Transmission Electron Microscope (TEM)

Mouse cardiac tissue samples were fixed with glutaraldehyde and 1% osmium tetroxide, dehydrated through a graded series of acetone, and embedded in Epon‐812 resin. Ultrathin sections (70 nm) were mounted on copper grids and stained with uranyl acetate and lead citrate for 10 and 2 min, respectively. The sections were subsequently examined using a JEM‐1400FLASH transmission electron microscope (JEOL, Japan) to acquire images. For each group, myocardial tissue samples were obtained from 5 mice. From each sample, three non‐overlapping fields were randomly selected. Mitochondria were classified as damaged if they exhibited any of the following ultrastructural criteria: marked swelling, reduced matrix electron density, disrupted or absent cristae, vacuolization, or membrane discontinuity. Autophagosomes were identified as double‐membrane vacuoles containing cytoplasmic material/organelles. All analyses were performed in a double‐blind manner.

### 2.11 Flow Cytometry Analysis

Mitochondrial membrane potential (MMP) was assessed using a JC‐1 assay kit (C2003S, Beyotime). The JC‐1 working solution was prepared by diluting 50 μL of JC‐1 (200X) in 8 mL of ultrapure water, mixing thoroughly, and then adding JC‐1 Staining Buffer (5X). For JC‐1 staining of NMCMs, 600,000 cells were resuspended in 0.5 mL of cell culture medium. Then, 0.5 mL of the JC‐1 working solution was added to the cell suspension, followed by incubation at 37°C for 20 min in the dark. The cells were centrifuged at 600× *g* for 3 min at 4°C, and the supernatant was carefully aspirated. The cell pellet was resuspended in 1 mL of JC‐1 Staining Buffer (1X) and centrifuged again at 600× *g* for 5 min at 4°C. After discarding the supernatant, the cells were resuspended in an appropriate volume of JC‐1 Staining Buffer (1X). MMP was then immediately analyzed based on JC‐1 staining using a flow cytometer (A00‐1‐1102, Beckman). JC‐1 exists as a monomer in the cytoplasm, emitting green fluorescence (excitation/emission: 514/529 nm). When the mitochondrial membrane potential increases, JC‐1 forms aggregates that emit red fluorescence (excitation/emission: 585/590 nm). MMP was expressed as the ratio of red to green fluorescence intensity.

For the detection of mitochondrial reactive oxygen species (ROS), we utilized the MitoSOX Red reagent kit (S0061S, Beyotime, China). According to the manufacturer's protocol, NMCMs were incubated with 200 μL of the prepared MitoSOX Red working solution for 20 min at 37°C. After washing three times with serum‐free medium, the cells were resuspended and analyzed by flow cytometry.

### 2.12 Reverse Transcription Quantitative Polymerase Chain Reaction (RT‐qPCR)

Total RNA was extracted from NMCMs using Trizol reagent. Messenger RNA (mRNA) was then reverse‐transcribed into complementary DNA (cDNA) using a commercial reverse transcription kit (CW2569, CWBIO, China). Quantitative real‐time polymerase chain reaction (RT‐qPCR) was performed on a QuantStudio 1 real‐time PCR system (Applied Biosystems, USA) using the UltraSYBR Mixture (CW2601, CWBIO, China). The thermal cycling conditions were as follows: initial pre‐denaturation at 95°C for 10 min, followed by 40 cycles of denaturation at 95°C for 15 s and annealing/extension at 65°C for 30 s. The relative mRNA expression levels of the target genes were calculated using the 2^−ΔΔCt^ method, with GAPDH serving as the internal reference gene. The primer sequences for all genes are listed in Table 2.

**TABLE 2:** Primer sequences information.

Primer name	Sequence‐Forward (5′‐3′)	Sequence‐Reverse (5′‐3′)
*GADPH*	GCGACTTCAACAGCAACTCCC	CACCCTGTTGCTGTAGCCGTA
*CFTR*	GGACTCTGGACACTTCGAGC	CATTTGGAACCAGCGCAAGG
*USP45*	GTCATGTGTGAAGAATGTGCGA	TGACTTAGTTGGTTCCTTTCTTCT
*USP10*	TTGGTCCTCAAGGGTACAGG	TAGCTTGTTTTACCTGGCATTGG
*USP33*	TTCGGCTCAACAGAGCATTTC	TGGACCTGGGGGATCCTTT
*USP9X*	CGGGATCGAGCTTTTGCATC	AACCAAACACAACCCGGGAG

### 2.13 Co‐Immunoprecipitation (CO‐IP) and IP

Protein was extracted from NMCMs using RIPA lysis buffer and aliquoted into Input, IP, and IgG tubes. The Input sample was processed normally for subsequent Western blot analysis. The IP and IgG tubes were incubated overnight at 4°C with a primary antibody against CFTR and control IgG antibody, respectively, followed by conjugation with Protein A/G agarose magnetic beads. After co‐immunoprecipitation, the samples were centrifuged at 3000 rpm for 3 min at 4°C. The resulting pellets were subjected to western blotting using antibodies against USP45 or ubiquitin (Ub).

### 2.14 Seahorse Assay

The mitochondrial oxygen consumption rate (OCR) of NMCMs was measured using an XFe96 Flux Analyzer (Seahorse Biosciences, Agilent). NMCMs (5 × 10^3^ cells/well) were seeded in XF‐96 plates with 80 μL DMEM and incubated overnight at 37°C. The mitochondrial stress test was performed by sequential addition of oligomycin (1.5 μM), carbonyl cyanide‐4‐(trifluoromethoxy)phenylhydrazone (FCCP, 1.0 μM), and rotenone/antimycin A (each 0.5 μM). Hoechst dye was used for nuclear staining, and OCR values were normalized to cell count using a multi‐mode microplate reader (MB‐530, HEALES).

### 2.15 Cell Counting Kit‐8 (CCK‐8) Assay

NMCMs were seeded into 96‐well plates at a density of 5 × 10^3^ cells per well in 100 μL of DMEM and incubated at 37°C in a 5% CO_2_ incubator until cell attachment. After the indicated treatments, 10 μL of CCK‐8 reagent (C0038, Beyotime, China) was added to each well, and the plates were further incubated for 4 h. The absorbance of each well was measured at 450 nm using a microplate reader.

### 2.16 5‐Ethynyl‐2′‐Deoxyuridine (EDU) Assay

NMCMs were inoculated at 5 × 10^3^ cells per well. Subsequently, cells were incubated with EDU (50 μM, C0078S, Beyotime, China) overnight at 37°C. After EDU incorporation, cells were fixed with 4% paraformaldehyde and permeabilized with 0.5% Triton X‐100. Nuclei were counterstained with Hoechst 33342. The percentage of EDU‐positive cells was quantified using a fluorescence microscope.

### 2.17 Apoptosis Detection

NMCMs were seeded into 96‐well plates at a density of 5 × 10^3^ cells per well in 100 μL of culture medium. After the indicated treatments, cells were digested with EDTA‐free trypsin and collected. The cells were then resuspended in 500 μL of Binding Buffer, followed by the addition of 5 μL of Annexin V‐APC (KGA1030, KeyGEN BioTECH, China) and 5 μL of propidium iodide (PI, MB2920, Meilun Bio, China). The mixture was gently vortexed and incubated for 10 min at room temperature in the dark. The apoptosis rate was immediately analyzed using a flow cytometer.

### 2.18 Data Analysis

Continuous data are reported as mean ± standard deviation. After verifying the assumptions of normality (using the Shapiro–Wilk test) and homogeneity of variances (using Bartlett's test), as appropriate, intergroup comparisons were made. Specifically, differences between two groups were analyzed by an unpaired Student's *t*‐test, while differences among more than two groups were analyzed by one‐way analysis of variance (ANOVA), followed by Tukey's honest significant difference test for post hoc comparisons. All statistical analyses were carried out with GraphPad Prism, version 8.0. Unless otherwise stated, a two‐tailed *p*‐value < 0.05 was considered to indicate statistical significance.

## 3 Results

### 3.1 The Aging of Myocardial Cells in AF Patients Is Accompanied by a Decrease in CFTR Levels

**FIGURE 1:**
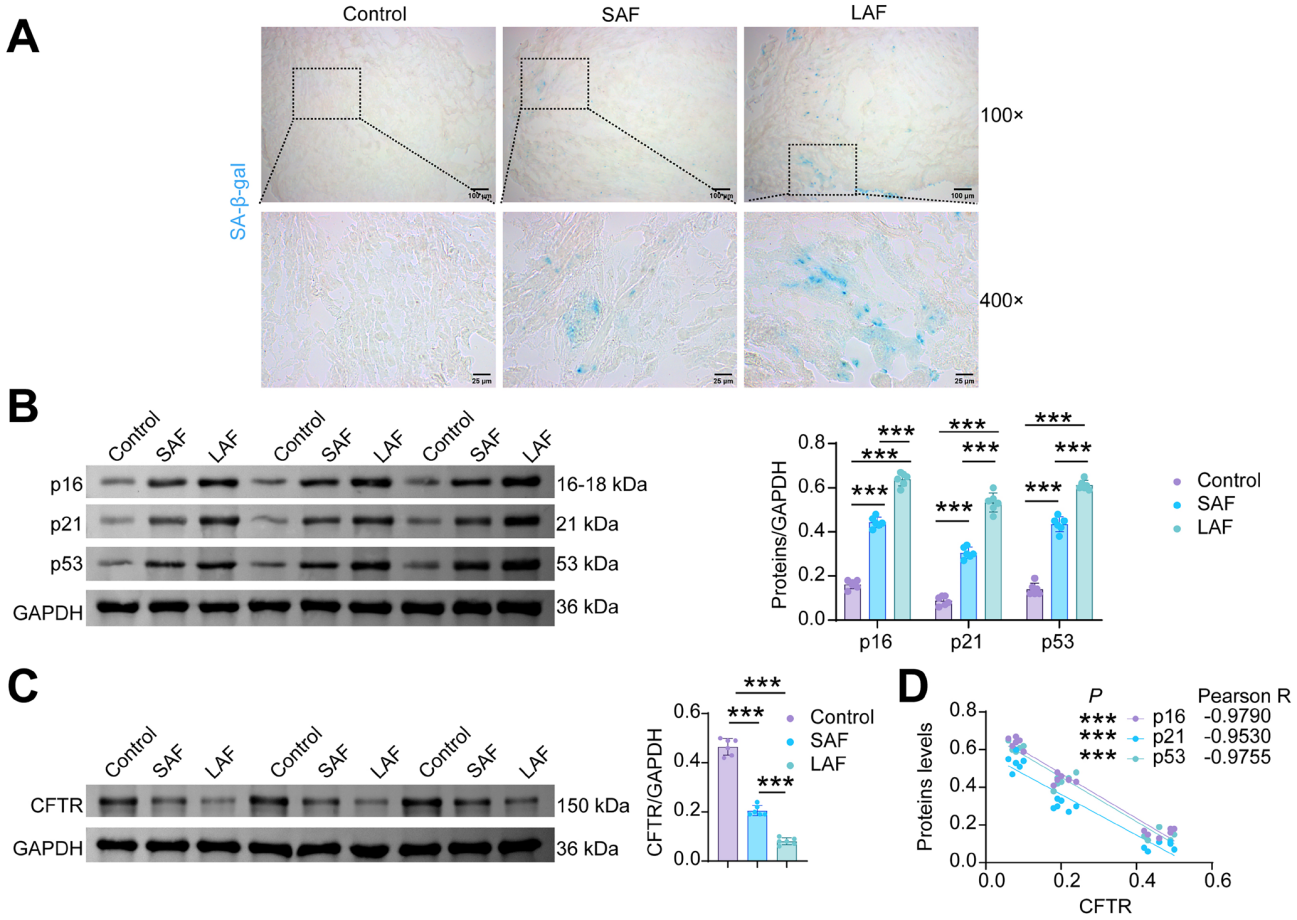
Increased senescence markers and reduced CFTR expression in LAA of AF patients. (A) SA‐β‐gal staining. (B) WB analysis of p16, p21 and p53. (C) WB analysis of CFTR. (D) Pearson correlation analysis was performed to examine the correlations of CFTR with p16, p21, and p53 separately. *n* = 6, ***p < 0.001.

Aging is a major risk factor for AF, as senescence of atrial cardiomyocytes increases susceptibility to AF (Jansen et al. 2025). SA‐β‐gal staining is currently the most commonly used and classic indicator for identifying cellular senescence. Several proteins associated with the senescence‐associated secretory phenotype (SASP) in cardiomyocyte senescence—including p16, p21, and p53 (Redgrave et al. 2023). We assessed senescence markers in left atrial appendage (LAA) tissue obtained during open‐heart surgery from patients with normal sinus rhythm (Control group), short‐term atrial fibrillation (SAF), and long‐standing permanent atrial fibrillation (LAF). Compared to the Control group, expression of the senescence markers SA‐β‐gal (Figure 1A), p16, p21, and p53 (Figure 1B) was significantly elevated in the LAA tissue of both SAF and LAF patients. This increase was more pronounced in LAF patients than in SAF patients. Additionally, we found that cystic fibrosis transmembrane conductance regulator (CFTR) expression was reduced in the LAA tissue of AF patients, with LAF patients exhibiting lower CFTR levels than SAF patients (Figure 1C). Pearson correlation analysis revealed a negative correlation between CFTR expression levels and p16, p21, and p53 levels in atrial tissue (Figure 1D). These results indicate that the hearts of AF patients exhibit features of senescence, concomitant with downregulation of CFTR.

### 3.2 CFTR Reduction Occurs Alongside Mitochondrial Damage in Cardiomyocytes of D‐Galactose‐Induced Senescent Mice

**FIGURE 2:**
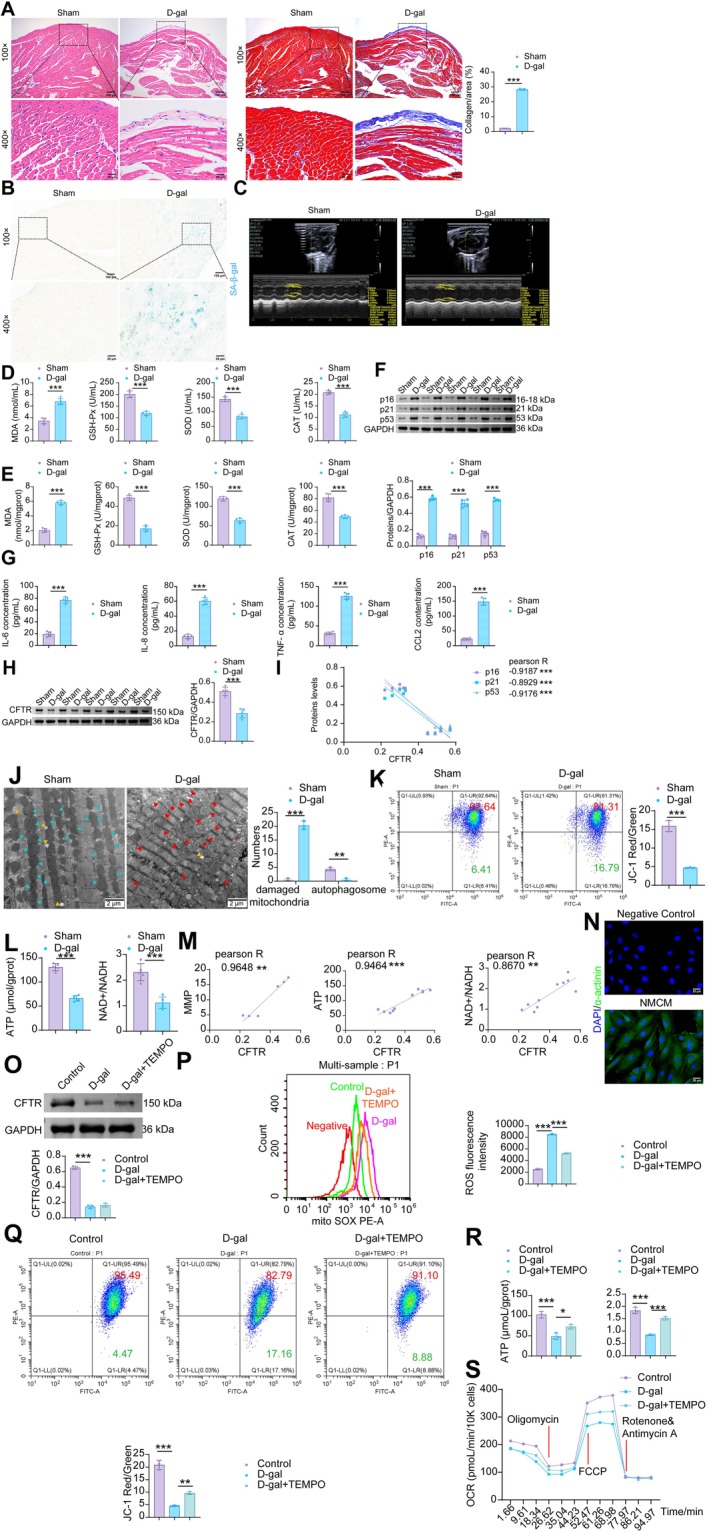
The decrease in CFTR in cardiac myocytes coincides with elevated mitochondrial oxidative stress in the aging murine heart. (A) H&E and Masson staining of mouse myocardial tissue. (B) SA‐β‐gal staining in mouse myocardial tissue. (C) Echocardiography examination. (D) The levels of MDA, SOD, GSH‐Px and CAT in the peripheral blood of mice. (E) The levels of MDA, SOD, GSH‐Px and CAT in the mouse myocardial tissue. (F) The protein levels of p16, p21 and p53 in the mouse myocardial tissue. (G) The levels of SASP factors (IL‐6, IL‐8, TNF‐α, and CCL2) in the mouse myocardial tissue. (H) The protein levels of CFTR in the mouse myocardial tissue. (I) Pearson Correlation Analysis of CFTR with p53, p21, and p16 Expression. (J) Assessment of mitochondrial damage in mouse CMs by TEM. (K) Flow cytometric JC‐1 staining for the determination of MMP in mouse myocardial tissue. (L) ATP and NAD^+^/NADH levels in mouse myocardial tissue. (M) Pearson correlation analysis of CFTR with ATP and NAD^+^/NADH levels in mouse myocardial tissue. (O) IF staining of α‐actinin for extracting NMCMs. (N) WB analysis of CFTR in NMCMs. (P) Flow cytometric analysis of mitochondrial ROS in NMCMs. (Q) Flow cytometric JC‐1 staining for the determination of MMP in NMCMs. (R) ATP and NAD+/NADH levels in NMCMs. (S) Mitochondrial oxygen consumption rate (OCR) levels in NMCMs were measured using an XFe96 Flux Analyzer. Data are presented as mean ± SD. The sample sizes were *n* = 5 for Figure 2A–M and *n* = 3 for Figure 2N–S. *p < 0.05, **p < 0.01, ***p < 0.001.

Next, we validated CFTR expression in the D‐galactose (D‐gal)‐induced cardiac aging mouse model. In the Sham group, myocardial tissue exhibited compactly arranged cardiomyocytes with normal cellular architecture. Compared to the Sham group, D‐gal‐induced mice exhibited observable signs of cardiac remodeling, characterized by apparently enlarged cardiomyocyte nuclei (qualitative observation), myofiber disarray, and increased collagen deposition (Figure 2A). Concurrently, SA‐β‐gal activity was elevated in the myocardial tissue of the model mice (Figure 2B). Echocardiography revealed that D‐gal induction led to reduced ejection fraction (EF), fractional shortening (FS) percentages, left ventricular anterior wall thickness at end‐systole (LVAW;s), and left ventricular posterior wall thickness at end‐systole (LVPW;s), while left ventricular internal diameter at end‐systole (LVID;s), left ventricular internal diameter at end‐diastole (LVID;d), left ventricular volume at end‐diastole (LV Vol;d), and left ventricular mass (LV Mass) were increased (Figures 2C and S1). Relative to the Sham group, model mice exhibited elevated malondialdehyde (MDA) levels in both serum (Figure 2D) and myocardial tissue (Figure 2E), accompanied by decreased activities of superoxide dismutase (SOD), glutathione peroxidase (GSH‐Px), and catalase (CAT). D‐gal treatment increased the protein expression of p16, p21, and p53 (Figure 2F), as well as the levels of SASP factors (IL‐6, IL‐8, TNF‐α, and CCL2) in myocardial tissue (Figure 2G), whereas it decreased CFTR expression (Figure 2H). CFTR expression in mouse myocardium was negatively correlated with p16, p21, and p53 levels (Figure 2I). These results demonstrate that CFTR is downregulated in D‐gal‐induced aging mouse hearts, concurrent with an increase in senescence markers.

Reportedly, aberrant CFTR expression may be associated with disrupted mitochondrial antioxidant defense (Rubin et al. 2025). To further explore the relationship between CFTR and mitochondrial oxidative stress in mouse myocardial tissue, we conducted the following experiments. TEM analysis revealed a substantial increase in damaged mitochondria and a reduction in autophagosomes in cardiomyocytes after D‐gal induction (Figure 2J). Compared to the Sham group, the D‐gal group showed a decrease in MMP (Figure 2K), as well as reduced levels of ATP and NAD^+^/NADH (Figure 2L). Pearson correlation analysis demonstrated positive correlations between CFTR expression and MMP, ATP, and NAD^+^/NADH levels (Figure 2M). Isolated neonatal mouse cardiomyocytes (NMCMs) were identified by positive α‐actinin immunofluorescence staining, confirming successful isolation and culture (Figure 2N). To determine the optimal time course for D‐gal‐induced cardiomyocyte senescence, we treated cardiomyocytes with D‐gal for 0, 1, 2, 5, and 10 days. The results showed that compared with the control group, cardiomyocytes exhibited decreased cell viability (Figure S2A) and proliferation (Figure S2B), increased apoptosis (Figure S2C), and upregulation of senescence‐associated proteins (p16, p21, and p53) as early as day 2 (Figure S2D). These senescent phenotypes became progressively more pronounced with longer treatment duration and were most evident at day 10. These findings indicate that although 48 h (day 2) of D‐gal treatment is sufficient to induce significant senescent changes in cardiomyocytes, the effect continues to strengthen over time. Therefore, we selected day 10, when the senescent phenotype was most robust, as the D‐gal modeling duration for our formal experiments. Compared to the Control group, D‐gal‐induced NMCMs exhibited decreased CFTR protein expression, which was not affected by treatment with the mitochondrial ROS‐specific antioxidant TEMPO (2,2,6,6‐Tetramethylpiperidin‐1‐oxyl) (Figure 2O). D‐gal induction resulted in increased ROS generation in NMCMs, which was partially attenuated by TEMPO (Figure 2P). In contrast, the D‐gal‐induced reductions in mitochondrial membrane potential (MMP), adenosine triphosphate (ATP), and the nicotinamide adenine dinucleotide (NAD^+^)/reduced nicotinamide adenine dinucleotide (NADH) ratio were partially reversed by TEMPO treatment (Figure 2Q,R). Additionally, we performed Seahorse analysis to assess cellular mitochondrial stress. Our findings showed that the oxygen consumption rate (OCR) in D‐gal‐induced NMCMs was significantly reduced under both basal respiration and maximal respiration conditions, while TEMPO partially restored the D‐gal‐impaired OCR (Figure 2S). These in vivo and in vitro results suggest that decreased CFTR expression in the aging heart may be positively associated with enhanced mitochondrial oxidative stress in cardiomyocytes.

### 3.3 CFTR Alleviates the Cardiac Aging Phenotype Induced by D‐Gal in Mice

**FIGURE 3:**
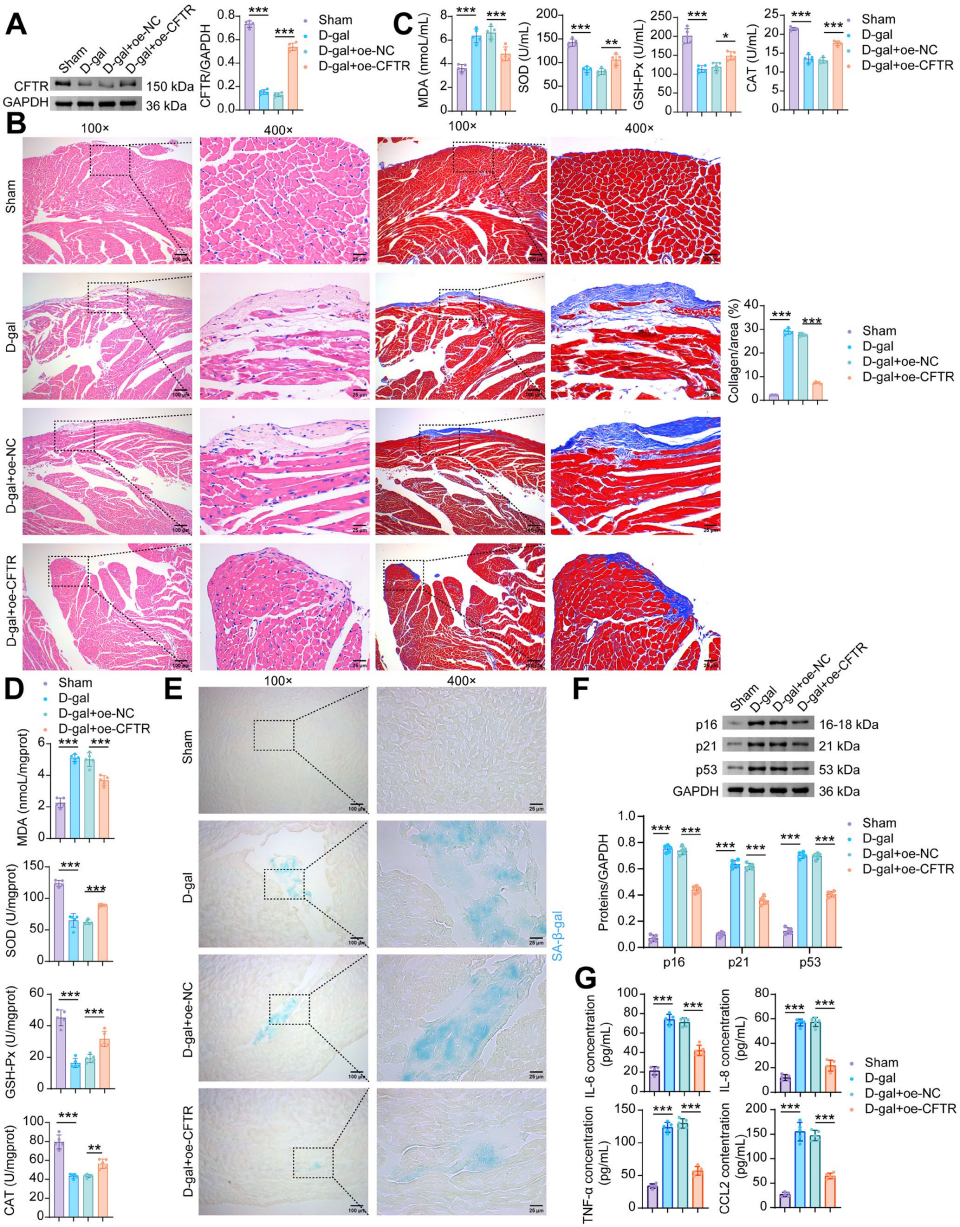
Overexpression of CFTR rescues mice from D‐galactose‐induced cardiac senescence. (A) WB analysis of CFTR. (B) H&E and masson staining. (C) The levels of MDA, SOD, GSH‐Px and CAT in the serum of mice. (D) The levels of MDA, SOD, GSH‐Px and CAT in the mouse myocardial tissue. (E) SA‐β‐gal staining of the mouse myocardial tissue. (F) The levels of p16, p21, and p53 proteins in the mouse myocardial tissue. (G) The levels of SASP factors (IL‐6, IL‐8, TNF‐α, and CCL2) in the mouse myocardial tissue. Data are presented as mean ± SD, *n* = 5, *p < 0.05, **p < 0.01, ***p < 0.001.

To investigate the role of CFTR in D‐galactose‐induced cardiac senescence, we examined the effect of CFTR overexpression on senescence‐related phenotypes in mouse myocardial tissue. Compared with the D‐gal+oe‐NC group, CFTR overexpression not only elevated CFTR levels in cardiac tissue but also alleviated D‐gal‐induced disorganization of myocardial fibers and excessive collagen deposition (Figure 3A,B). Lentivirus‐mediated delivery of oe‐CFTR partially reversed the D‐gal‐induced increase in MDA and the decrease in SOD, GSH‐Px, and CAT activities in both peripheral blood and myocardial tissue (Figure 3C,D). Furthermore, CFTR overexpression reduced the elevated levels of senescence‐associated markers, including SA‐β‐gal, p16, p21, and p53, in cardiac tissues of D‐gal‐treated mice (Figure 3E,F). CFTR overexpression partially reversed the D‐gal‐induced elevation of SASP factors (IL‐6, IL‐8, TNF‐α, CCL2) in mouse serum (Figure 3G). These results demonstrate that CFTR overexpression attenuates D‐gal‐induced cardiomyocyte senescence.

### 3.4 CFTR Protects Cardiomyocytes From D‐Gal‐Induced Oxidative Stress and Senescence

**FIGURE 4:**
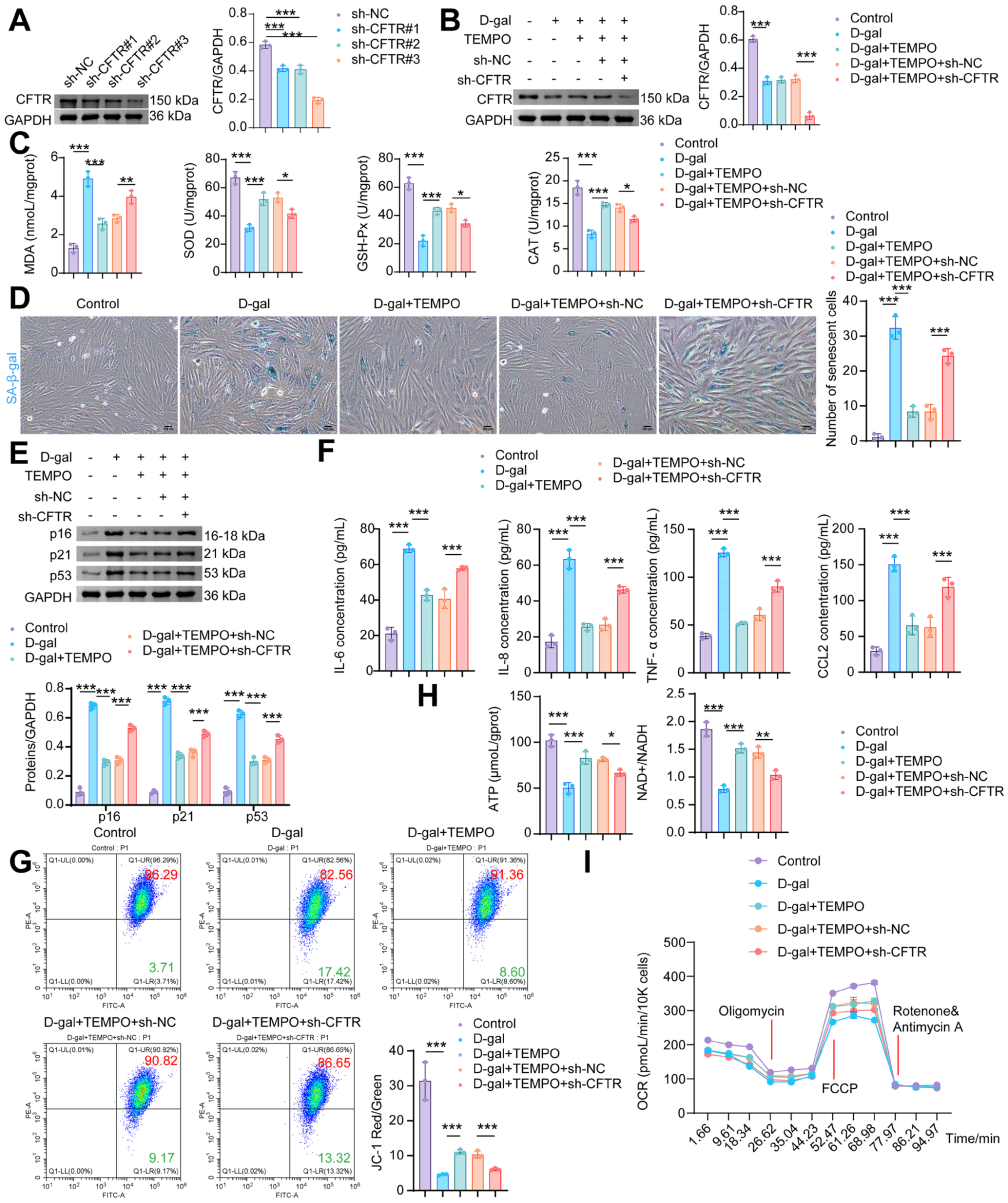
CFTR inhibition aggravates D‐gal‐induced cardiomyocyte senescence and oxidative stress. (A, B) WB analysis of CFTR. (C) MDA, SOD, GSH‐Px, and CAT levels in NMCMs. (D) SA‐β‐gal staining. (E) WB analysis of p16, p21, and p53. (F) The levels of SASP factors (IL‐6, IL‐8, TNF‐α, and CCL2) in NMCMs. (G) MMP was assessed using JC‐1 staining followed by flow cytometry. (H) Levels of ATP, NAD^+^/NADH of NMCMs. (I) Mitochondrial OCR levels in NMCMs were measured using an XFe96 Flux Analyzer. Data are presented as mean ± SD, *n* = 3, *p < 0.05, **p < 0.01, ***p < 0.001.

Deprivation of wild‐type CFTR in brain, heart, and lung tissues has been associated with inflammation and oxidative stress, and mutations in CFTR render mitochondria more susceptible to damage (Scialò et al. 2024; Wu et al. 2023). To further investigate the role of CFTR in D‐gal‐induced cardiomyocyte senescence and mitochondrial oxidative stress in vitro, we transfected cells with sh‐CFTR (#1, #2, #3), which resulted in reduced CFTR expression levels (Figure 4A). The most effective knockdown construct, sh‐CFTR#3, was selected for subsequent experiments. Following D‐gal stimulation, CFTR expression was decreased in NMCMs. TEMPO, a superoxide dismutase mimetic, had no effect on CFTR expression. Compared with the D‐gal+TEMPO+sh‐NC group, transfection with sh‐CFTR further reduced CFTR expression in NMCMs (Figure 4B). TEMPO treatment reduced the D‐gal‐induced increase in MDA levels in NMCMs, while CFTR knockdown partially counteracted this effect of TEMPO. The decreased levels of SOD, GSH‐Px, and CAT induced by D‐gal were elevated via TEMPO; however, CFTR knockdown partly reversed the protective effects of TEMPO (Figure 4C). The positive rate of SA‐β‐gal staining was increased in D‐gal‐induced NMCMs. TEMPO reduced the increased SA‐β‐gal positive staining rate in D‐gal‐stimulated NMCMs. Inhibition of CFTR partially reversed the effect of TEMPO (Figure 4D). Concurrently, CFTR knockdown elevated the expression of p16, p21, and p53, which were upregulated in D‐gal‐induced NMCMs but reduced by TEMPO (Figure 4E). TEMPO treatment reduced the D‐gal‐induced secretion of SASP factors (IL‐6, IL‐8, TNF‐α, and CCL2) in NMCMs, whereas transfection with sh‐CFTR partially reversed the effects of TEMPO (Figure 4F). Regarding mitochondrial function, the MMP of NMCMs decreased upon D‐gal stimulation. Compared to the D‐gal group, the addition of TEMPO increased the MMP of NMCMs, and this effect was attenuated by sh‐CFTR (Figure 4G). Furthermore, TEMPO increased the reduced ATP and NAD+/NADH levels in D‐gal‐induced NMCMs, and these increases were reversed by CFTR knockdown (Figure 4H). Notably, TEMPO treatment partially rescued the D‐gal‐reduced OCR, whereas knockdown of CFTR reversed these protective effects (Figure 4I). These results suggest that CFTR may be involved in alleviating D‐gal‐induced cardiomyocyte senescence and mitochondrial oxidative stress.

### 3.5 CFTR Alleviates Mitochondrial Oxidative Stress Damage by Enhancing PMCA Activity to Reduce Cytoplasmic Ca^2+^ Levels

**FIGURE 5:**
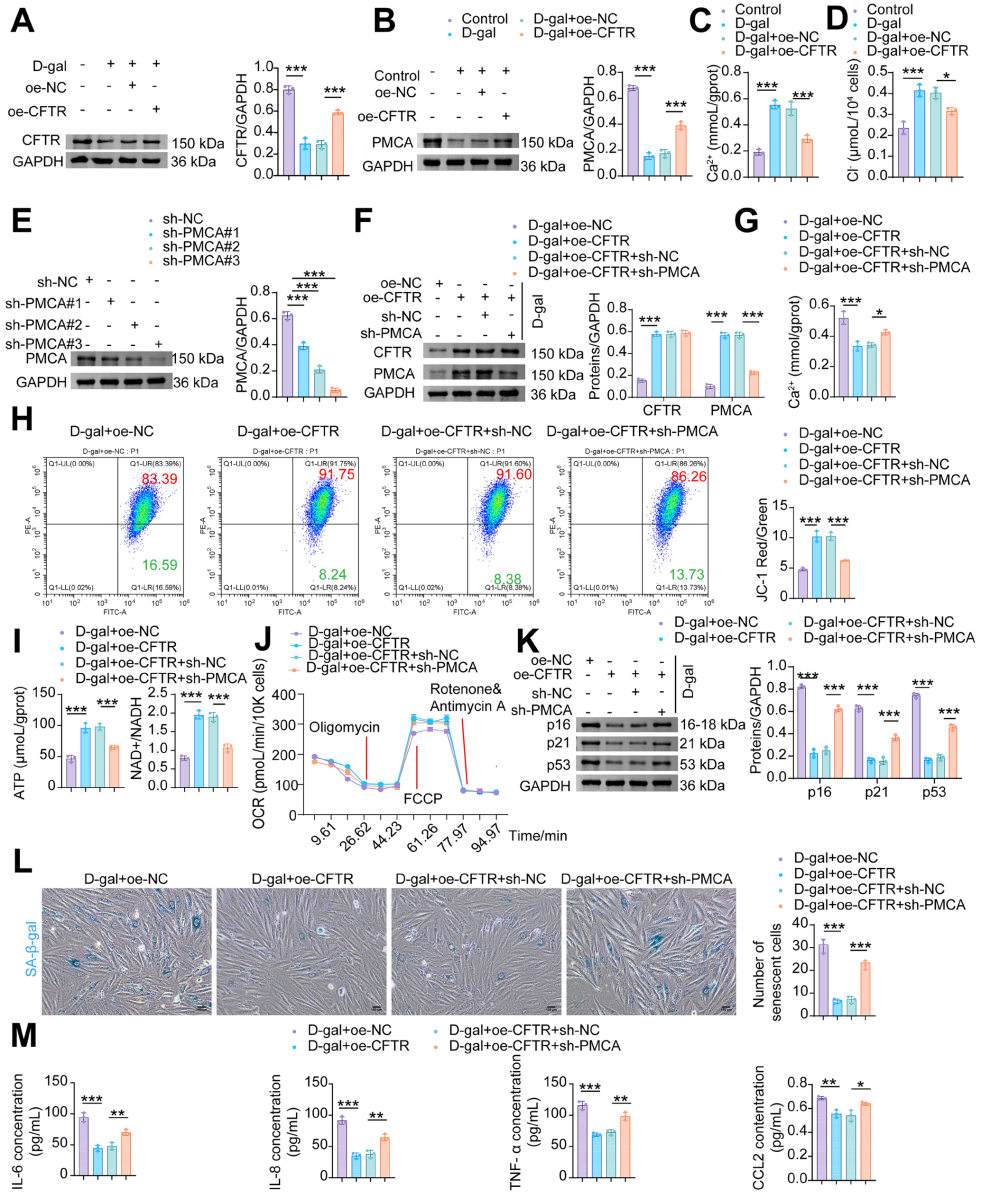
PMCA‐mediated Ca^2^+ clearance is required for CFTR to ameliorate mitochondrial dysfunction and cellular senescence in D‐gal‐treated NMCMs. (A) WB analysis of PMCA. (B) Levels of Ca^2+^. (C) Levels of Cl^−^. (D) WB analysis of PMCA. (E) WB analysis of CFTR and PMCA. (F) Levels of Ca^2+^. (G) Flow cytometric analysis of MMP with JC‐1. (H) Levels of ATP and NAD^+^/NADH. (I) SA‐β‐gal staining was performed to detect senescent cells. (J) Mitochondrial OCR levels in NMCMs were measured using an XFe96 Flux Analyzer. (K) WB analysis of p16, p21, and p53. (L) SA‐β‐gal staining in NMCMs. (M) The levels of SASP factors (IL‐6, IL‐8, TNF‐α, and CCL2) in NMCMs. Data are presented as mean ± SD, *n* = 3, *p < 0.05, **p < 0.01, ***p < 0.001.

Previous research has demonstrated that reduced CFTR expression impairs PMCA function, leading to sustained cytosolic Ca^2+^ elevation in ethanol‐treated mouse and human pancreatic organoids (Madácsy et al. 2022). In NMCMs, overexpression of CFTR counteracted the D‐gal‐induced downregulation of PMCA expression (Figure 5A). CFTR upregulation also reduced the D‐gal‐elevated levels of cytosolic Ca^2+^ and Cl^−^ (Figure 5B,C). Next, we transfected D‐gal‐induced NMCMs with sh‐CFTR to examine the effect of CFTR knockdown on PMCA expression and its mediated calcium homeostasis. Transfection with sh‐CFTR further exacerbated the D‐gal‐induced downregulation of CFTR and PMCA protein expression in NMCMs (Figure S3A,B), as well as the elevations of intracellular Ca^2+^ and Cl^−^ (Figure S3C,D). Compared to the sh‐NC group, all sh‐PMCA constructs (#1, #2, #3) significantly knocked down PMCA expression (Figure 5D). Among these, sh‐PMCA#3, which exhibited the most potent silencing effect, was selected for subsequent experiments. Transfection with oe‐CFTR in D‐gal‐treated NMCMs increased the expression of both CFTR and PMCA compared to the D‐gal+oe‐NC group (Figure 5E) and reduced intracellular Ca^2+^ accumulation (Figure 5F). In contrast, compared to the D‐gal+oe‐CFTR+sh‐NC group, knockdown of PMCA did not affect CFTR expression but specifically reduced PMCA protein levels (Figure 5E), and concurrently increased intracellular Ca^2+^ (Figure 5G). Furthermore, PMCA knockdown abolished the recovery of mitochondrial membrane potential (MMP) achieved by CFTR overexpression (Figure 5H). CFTR overexpression in D‐gal‐treated NMCMs increased ATP production, the NAD^+^/NADH ratio (Figure 5I), and OCR (Figure 5J), downregulated the protein expression of senescence markers p16, p21, and p53 (Figure 5K), decreased the percentage of SA‐β‐gal‐positive cells (Figure 5L), and reduced the secretion of IL‐6, IL‐8, TNF‐α, and CCL2 (Figure 5M). Importantly, inhibition of PMCA largely reversed these protective effects mediated by CFTR overexpression (Figure 5G–M). These data indicate that CFTR mitigates mitochondrial impairment and cellular senescence via a PMCA‐mediated reduction in cardiomyocyte Ca^2+^ accumulation.

### 3.6 USP45 Prevents the Degradation of CFTR by Inhibiting Its Ubiquitination

**FIGURE 6:**
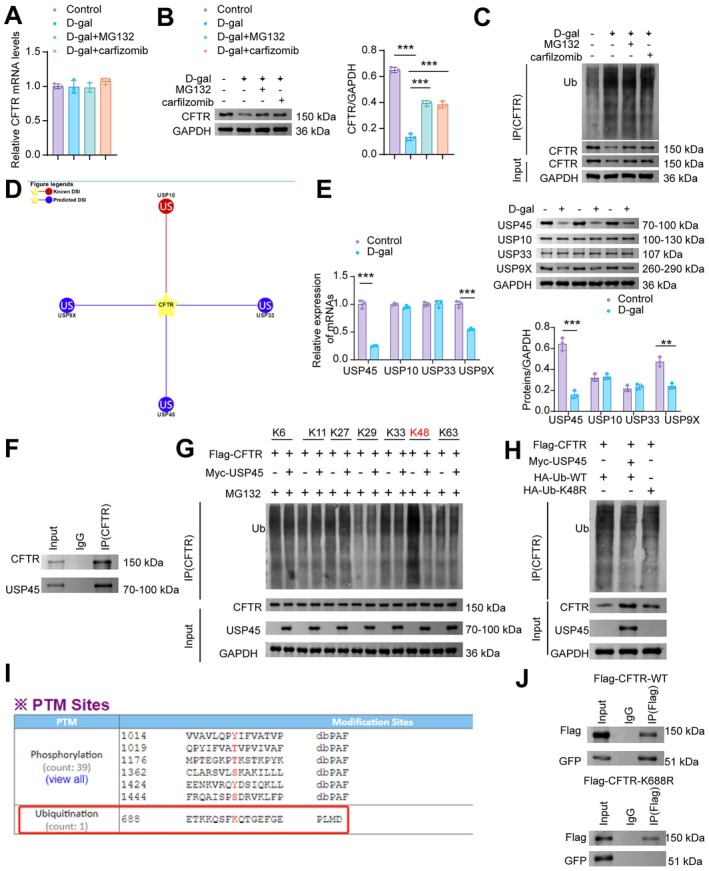
CFTR is deubiquitinated by USP45 at the K688 residue and on K48‐linked ubiquitin chains. (A) RT‐qPCR analysis of CFTR mRNA. (B) WB analysis of CFTR. (C) D‐gal‐induced NMCMs were treated with MG132 (10 μM for 12 h) and carfilzomib (1 μM for 12 h), followed by immunoprecipitation (IP) to assess the ubiquitination level of CFTR. (D) Prediction of CFTR‐targeting deubiquitinating enzymes was performed using the UbiBrowser 2.0 database. (E) RT‐qPCR and WB analysis of USP45, USP10, USP33, USP9X. (F) CO‐IP of CFTR and USP45. (G) Identification of USP45‐targeted ubiquitin linkages on CFTR by IP/WB. (H) Overexpression of USP45 or mutation of the ubiquitin chain residue to K48R (K48R mutant) resulted in the suppression of CFTR ubiquitination. (I) The GPS‐PTMD database predicted K688 as a potential ubiquitination site on CFTR. (J) NMCMs were co‐transfected with GFP‐USP45 and either WT or K688R CFTR fragments for 48 h. Proteins were pulled down using IgG and an anti‐CFTR antibody, and further analyzed by WB with antibodies against CFTR and USP45 (GFP). Data are presented as mean ± SD, *n* = 3, **p < 0.01, ***p < 0.001.

Inhibition of ubiquitination to stabilize the CFTR protein has long been recognized as a therapeutic target for various diseases, such as cystic fibrosis and kidney injury (Hou et al. 2024; Okiyoneda et al. 2024). We found that D‐galactose (D‐gal) and the proteasome inhibitors MG132 and carfilzomib did not affect CFTR mRNA expression (Figure 6A). However, MG132 and carfilzomib increased CFTR protein expression, which was reduced by D‐gal (Figure 6B), and attenuated D‐gal‐enhanced CFTR ubiquitination (Figure 6C). Subsequently, we queried the UbiBrowser 2.0 database (http://ubibrowser.bio‐it.cn/) and identified known deubiquitinating enzymes of CFTR, including USP10, as well as predicted ones such as USP9X, USP33, and USP45 (Figure 6D). Experimental validation showed that, compared with the control group, the expression of USP45 and USP9X was downregulated in D‐gal‐induced NMCMs, while the expression of USP10 and USP33 remained unchanged (Figure 6E). Among these, USP45, which exhibited the most significant downregulation, was selected for further investigation. Co‐immunoprecipitation (Co‐IP) results revealed a specific interaction between CFTR and USP45 proteins (Figure 6F). Under pathological conditions of aging, the downregulation of USP45 is a key factor leading to reduced CFTR protein levels in NMCMs.

To elucidate the mechanism by which USP45 mediates CFTR deubiquitination, we examined the types of ubiquitin chains (e.g., K48‐linked, K63‐linked) attached to CFTR and the modification levels at its ubiquitination sites after USP45 overexpression. Overexpression of USP45 significantly reduced ubiquitination at the K48 site of CFTR (Figure 6G). Compared with transfection of NMCMs with HA‐tagged wild‐type ubiquitin (HA‐Ub‐WT), both HA‐tagged K48‐only mutant ubiquitin (HA‐Ub‐K48R) and USP45 overexpression plasmids decreased CFTR ubiquitination levels and enhanced CFTR protein stability (Figure 6H). The GPS‐PTMD database predicted K688 as a ubiquitination site of CFTR (http://ptmd.biocuckoo.org/finalpage.php?uniprot=P13569) (Figure 6I). After successful transfection of cardiomyocytes with GFP‐tagged USP45, USP45 was unable to bind to the K688R CFTR mutant, unlike wild‐type CFTR (WT‐CFTR) (Figure 6J). These results demonstrate that USP45 specifically targets the K688 site of CFTR and removes K48‐linked polyubiquitin chains, thereby counteracting CFTR degradation via the proteasome pathway and enhancing its stability.

### 3.7 USP45 Alleviates Mitochondrial Oxidative Stress Damage and Cellular Senescence in Cardiomyocytes by Enhancing the Stability of the CFTR Protein

**FIGURE 7:**
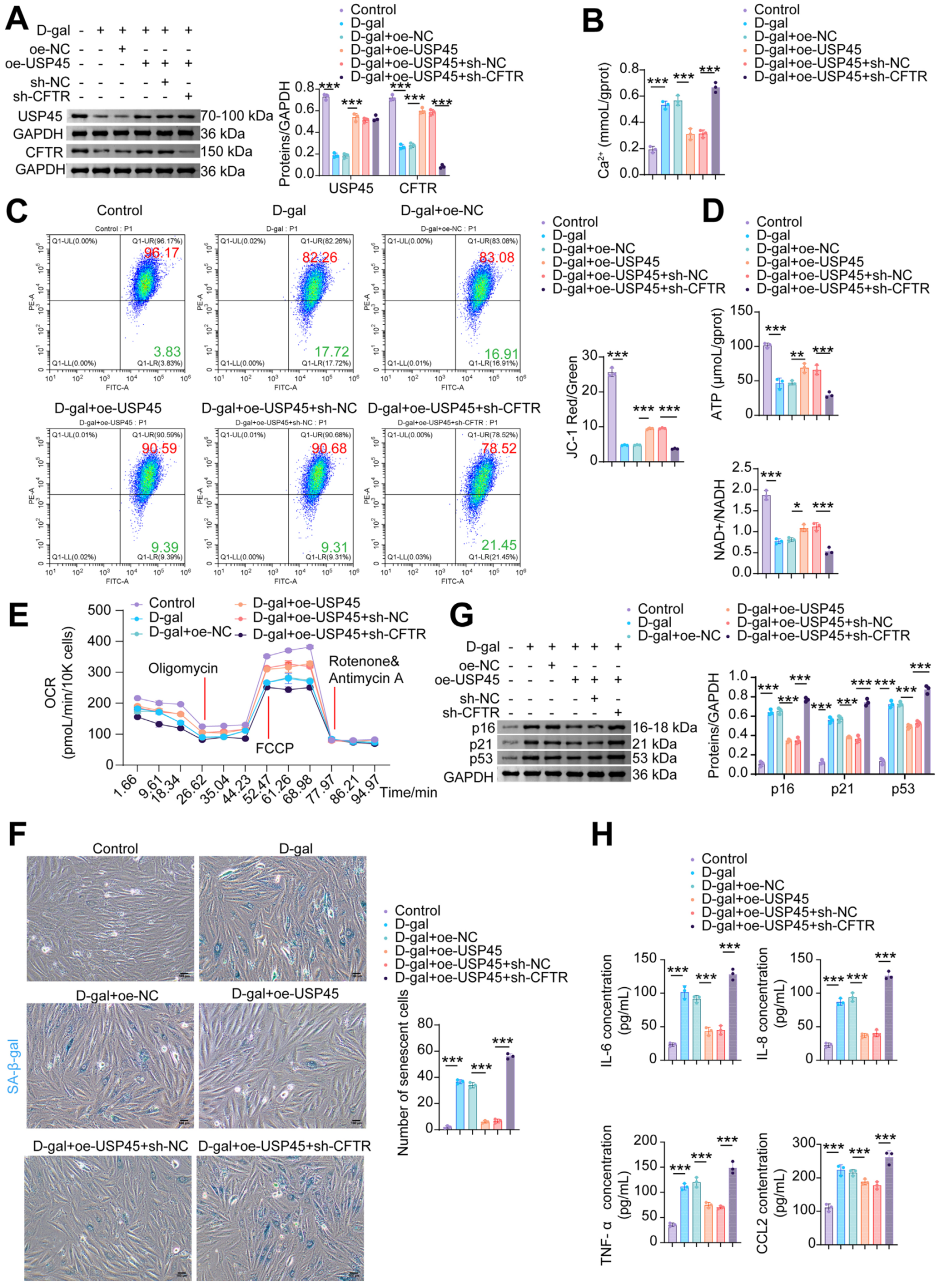
USP45 attenuates mitochondrial damage and senescence in CMs by upregulating CFTR protein stability. (A) WB analysis of USP45 and CFTR. (B) The Ca^2+^ level in NMCM. (C) MMP was measured by JC‐1 flow cytometry. (D) ATP and NAD^+^/NADH levels. (E) Mitochondrial OCR levels in NMCMs were measured using an XFe96 Flux Analyzer. (F) SA‐β‐gal staining. (G) WB analysis of p16, p21, and p53. (H) The levels of SASP factors (IL‐6, IL‐8, TNF‐α, and CCL2) in NMCMs. Data are presented as mean ± SD, *n* = 3, *p < 0.05, **p < 0.01, ***p < 0.001.

In vitro, we further investigated whether USP45 alleviates mitochondrial oxidative stress and cellular senescence in cardiomyocytes by regulating CFTR protein stability. Compared to the D‐gal+oe‐NC group, transfection with oe‐USP45 upregulated both USP45 and CFTR expression in D‐gal‐treated NMCMs. Knockdown of CFTR downregulated its own expression but did not affect USP45 levels in NMCMs (Figure 7A). Overexpression of USP45 reduced intracellular Ca^2+^ levels (Figure 7B), restored the D‐gal‐induced loss of mitochondrial membrane potential (MMP) (Figure 7C), and increased the levels of ATP and the NAD^+^/NADH ratio, which were impaired by D‐gal treatment (Figure 7D). These beneficial effects were abolished by CFTR inhibition (Figure 7B–D). While USP45 overexpression partially restored the D‐gal‐suppressed OCR, CFTR knockdown reversed the effect of oe‐USP45 compared with the D‐gal+oe‐USP45+sh‐NC group (Figure 7E). Furthermore, USP45 overexpression reduced the proportion of senescent NMCMs induced by D‐gal (Figure 7F) and decreased the protein expression of senescence markers p16, p21, and p53 (Figure 7G). Knockdown of CFTR reversed the anti‐senescence effects of USP45 overexpression (Figure 7F–G). Compared with the D‐gal+oe‐NC group, USP45 overexpression suppressed the secretion of IL‐6, IL‐8, TNF‐α, and CCL2 in NMCMs, whereas CFTR knockdown completely reversed the effect of oe‐USP45 (Figure 7H). These results demonstrate that USP45 attenuates mitochondrial damage and cellular senescence in cardiomyocytes by upregulating CFTR protein stability.

### 3.8 USP45 Stabilizes CFTR to Attenuate Cardiac Aging and Oxidative Stress

**FIGURE 8:**
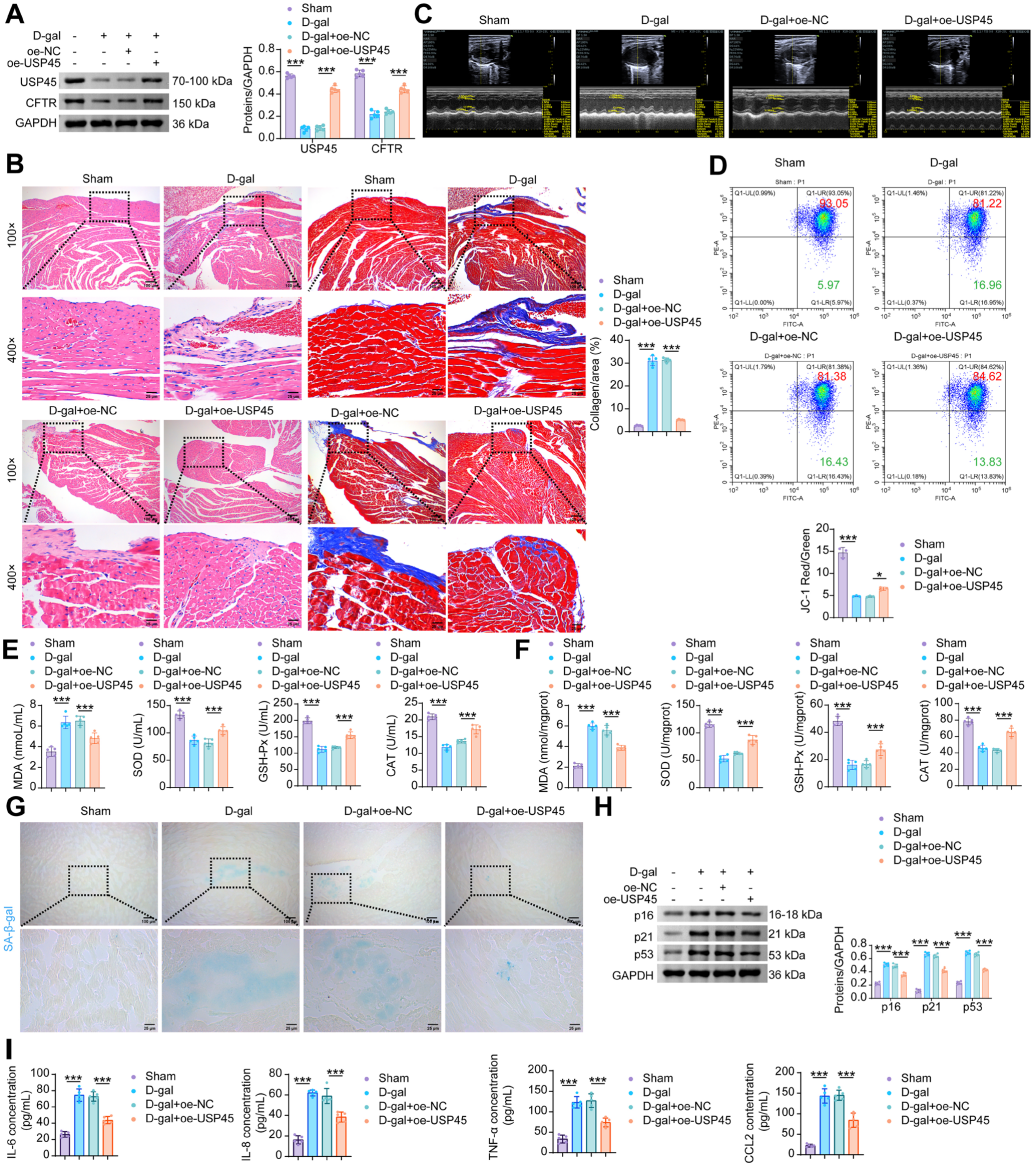
USP45 rescues cardiac aging and mitochondrial damage by mediating CFTR stabilization. (A) WB analysisi of USP45 and CFTR in the mouse myocardial tissue. (B) H&E and Masson staining of mouse myocardial tissue. (C) Echocardiography examination. (D) Flow cytometric JC‐1 staining for the determination of MMP in mouse myocardial tissue. (E) The levels of MDA, SOD, GSH‐Px and CAT in the serum of mice. (F) The levels of MDA, SOD, GSH‐Px and CAT in the mouse myocardial tissue. (G) SA‐β‐gal staining in mouse myocardial tissue. (H) The protein levels of p16, p21 and p53 in the mouse myocardial tissue. (I) The levels of SASP factors (IL‐6, IL‐8, TNF‐α, and CCL2) in the mouse myocardial tissue. Data are presented as mean ± SD, *n* = 5, *p < 0.05, ***p < 0.001.

In vivo, we further validated whether USP45 alleviates myocardial cell senescence and mitochondrial damage in mice via CFTR. Both USP45 and CFTR showed downregulation in D‐gal‐induced mice myocardial tissues. Compared with the D‐gal+oe‐NC group, USP45 overexpression reversed the reduced expression of both USP45 and CFTR in the myocardial tissues of D‐gal‐induced senescent mice (Figure 8A). Myocardial fibers in the control group were compactly and neatly arranged with clear structure, normal intercellular space, regular nuclear morphology, and centrally located nuclei. In contrast, the D‐gal group exhibited disordered myocardial tissue structure, including partially broken, dissolved, or necrotic myocardial fibers; swollen cardiomyocytes; and pyknotic or fragmented nuclei in some cells. Additionally, extensive diffuse blue collagen fiber deposition and significant fibrosis were observed in the myocardial interstitium, with a significantly higher collagen volume fraction compared to the control group. In comparison with the D‐gal+oe‐NC group, the D‐gal+oe‐USP45 group showed significant improvement in myocardial histopathological injury: myocardial fibers were more regularly arranged with reduced disruption, and cardiomyocyte swelling and necrosis were alleviated. Nuclear morphology was also more regular. Masson's trichrome staining further confirmed that USP45 overexpression markedly attenuated the degree of myocardial interstitial fibrosis and reduced collagen deposition in model mice compared to the D‐gal+oe‐NC group (Figure 8B). Echocardiography results demonstrated that USP45 overexpression partially reversed D‐gal‐induced alterations in cardiac function and ventricular structure. Specifically, it increased the D‐gal‐reduced EF, FS, LVAW;s, and LVPW;s, and decreased the D‐gal‐elevated LVID;s, LV Vol;s, LVID;d, LV Vol;d, and LV Mass (Figures 8C and S4). Furthermore, USP45 overexpression elevated the reduced mitochondrial membrane potential in senescent model mice (Figure 8D), decreased the D‐gal‐induced elevation of MDA levels in serum and myocardial tissue, and increased the D‐gal‐reduced levels of SOD, GSH‐Px, and CAT (Figure 8E,F). The positive rate of SA‐β‐gal staining in myocardial tissues of senescent model mice was reduced by oe‐USP45 (Figure 8G), and the protein expression of senescence markers p16, p21, and p53 was downregulated by USP45 overexpression (Figure 8H). USP45 overexpression partially inhibited the secretion of SASP factors elevated by D‐gal (Figure 8I). These results suggest that USP45 may alleviate mitochondrial damage and cellular senescence phenotypes in cardiomyocytes of aged mice by stabilizing the CFTR protein.

## 4 Discussion

Although the effects of aging have been extensively studied, the process of cardiomyocyte senescence remains poorly understood. Studies have shown that mitochondrial function and energy transduction efficiency are both impaired during the senescence of cardiomyocytes (Tepp et al. 2017). In the context of cardiac aging, alterations in metabolic substrates and enzyme activities lead to increased oxidative stress, elevated levels of ROS, and are accompanied by mitochondrial dysfunction and changes in gene expression (Hao and Liu 2023). This study reveals that USP45 alleviates D‐gal‐induced myocardial senescence and mitochondrial oxidative stress in mice by modulating the ubiquitination of CFTR.

The prevalence of AF increases with advancing age, and cardiomyocyte senescence is more pronounced in the AF population (Adili et al. 2022). This aligns with our findings, which indeed demonstrate more evident myocardial senescence in AF patients compared to healthy individuals. Furthermore, analysis of clinical samples in this study indicates that CFTR expression is downregulated in the myocardial tissue of AF patients and shows a negative correlation with senescence markers. CFTR deficiency has been shown to disrupt mitochondrial redox homeostasis and Ca^2+^/Cl^−^ balance (Jarosz‐Griffiths et al. 2024; Kleme et al. 2018). Studies indicate that mitochondrial dysfunction leads to insufficient ATP production and excessive ROS generation, which consequently disrupt intracellular Ca^2+^ homeostasis and sarcolemmal excitability in cardiomyocytes, ultimately triggering AF (He et al. 2025). Based on this evidence, we propose that downregulation of CFTR may be associated with exacerbated myocardial senescence and mitochondrial oxidative stress in AF patients.

Reduced CFTR expression has been implicated in skeletal muscle aging and mitochondrial oxidative stress (Chen et al. 2025). Concurrently, CFTR dysfunction may contribute to alterations in cardiac function (Duus et al. 2024). In our study, we observed that CFTR expression was indeed downregulated in the myocardial tissue of D‐gal‐induced aging mice. Notably, CFTR overexpression attenuated pathological damage in the hearts of these aging mice, alongside mitigating cardiomyocyte senescence phenotypes and mitochondrial oxidative stress. Sustained Ca^2+^ influx can lead to intracellular calcium overload, resulting in mitochondrial membrane damage, altered mitochondrial membrane potential, and reduced ATP production. The PMCA is responsible for extruding calcium ions from the cell (Chen et al. 2024). Importantly, CFTR‐dependent calmodulin recruitment determines PMCA4 activity (Madácsy et al. 2022). Our in vitro experiments demonstrated that inhibition of PMCA partially reversed the protective effects of CFTR overexpression on cardiomyocyte senescence and mitochondrial oxidative stress, leading to the re‐accumulation of Ca^2+^ within the cytoplasm that was otherwise extruded due to CFTR overexpression. These findings strongly suggest that CFTR is highly likely to restore Ca^2+^ homeostasis in senescent cardiomyocytes by activating PMCA, thereby alleviating mitochondrial damage and the senescent phenotype.

Deubiquitination refers to the process by which a series of deubiquitinating enzymes (DUBs) recognize ubiquitin‐tagged proteins and hydrolyze and remove ubiquitin chains from specific amino acid residues on substrate proteins (Zhan et al. 2024). Deubiquitination is considered as crucial as its counterpart, ubiquitination, in maintaining the half‐life, activity, and localization of proteins under both normal and pathological conditions (Bhattacharya et al. 2020). Deubiquitinating enzymes can stabilize the CFTR protein by removing polyubiquitin chains (Qian et al. 2025). In this study, we identified USP45 as the deubiquitinating enzyme for CFTR in cardiomyocytes, which specifically targets K48‐linked ubiquitin chains and modifies the K688 residue of CFTR. Knockdown of USP30 has been shown to alleviate D‐gal‐induced mitochondrial damage and inhibit cardiomyocyte senescence (Pan et al. 2021). Similarly, USP11 stabilizes murine double minute 2 (MDM2) in a manner dependent on its deubiquitinase activity, thereby suppressing cellular senescence (Kim et al. 2024). These studies indicate that deubiquitinating enzymes are involved in regulating cellular senescence, and their effects depend on their specific protein substrates. Both in vivo and in vitro experiments in this study demonstrate that USP45 can mitigate D‐gal‐induced cardiomyocyte senescence and mitochondrial oxidative stress damage by stabilizing CFTR.

Although this study reveals a novel mechanism through which USP45 stabilizes the CFTR protein by deubiquitinating it at the K688 residue and K48‐linked ubiquitin chains, thereby activating PMCA to restore calcium homeostasis and alleviate mitochondrial oxidative stress and cardiomyocyte senescence, certain limitations remain. The clinical sample size used in this study is relatively small, and further validation in a larger cohort study is needed. The D‐gal‐induced aging model, while classic, cannot fully replicate the multifactorial and complex process of natural human cardiac aging. Furthermore, the precise molecular mechanism of the interaction between CFTR and PMCA, and whether USP45 has additional targets in cardiac aging, requires further elucidation. The current intervention strategy of gene overexpression also remains distant from clinical translation. To address these issues, future research will first validate the generalizability of this pathway in various models, including naturally aged mice. We will employ techniques such as co‐immunoprecipitation and fluorescence resonance energy transfer (FRET) to dissect the formation mechanism of the CFTR‐PMCA complex. Concurrently, we will focus on screening small‐molecule compounds or gene therapy strategies that can specifically activate the USP45‐CFTR pathway, assessing their cardiac specificity and long‐term safety. Finally, by analyzing the correlation between the expression of USP45 and CFTR and cardiac function in human heart tissues across different age groups, we aim to translate these findings towards clinical application, providing a new theoretical foundation and potential therapeutic targets for delaying cardiac aging. Given the potential variability in TEM imaging planes, future research should utilize quantitative software for myofiber orientation analysis to achieve a more objective evaluation of myofiber organization. Furthermore, future studies should incorporate morphometric analysis to systematically quantify cardiomyocyte cross‐sectional area, thereby enabling a more comprehensive assessment of the relationship between CFTR regulation and cardiac structural remodeling.

## 5 Conclusion

In summary, this study demonstrates that USP45 enhances CFTR protein stability via deubiquitination, thereby activating PMCA to alleviate D‐gal‐induced mitochondrial oxidative stress and cardiomyocyte senescence. These findings provide a novel therapeutic strategy for intervening in cardiovascular diseases associated with cardiac aging.

## Author Contributions

**Chun Chen, Longtan Jiang, Yuewen Qiu, Chong Liu:** data curation, formal analysis, investigation, methodology, validation, visualization, writing – original draft, writing – review and editing. **Chun Chen:** data curation, formal analysis. **Xiao Long,** **Pengcui Wu, Liang Li, Haixia Xu:** data curation. **Li Yang:** data curation, resources, writing – review and editing. **Ping Deng:** resources, supervision, writing – review and editing. **Haixia Xu:** investigation, resources writing – review and editing. **Qiao Jin:** conceptualization, data curation, project administration, resources, supervision, writing – review and editing.

## Funding

The work was supported by Natural Science Foundation of Hunan Province (Nos. 2025JJ80555, 2026JJ30184, 2025JJ80562, 2026JJ81629) and Natural Science Foundation of Changsha City (No. kq2502323).

## Conflicts of Interest

The authors declare no conflicts of interest.

## Supporting information


**Figure S1:** Analysis of echocardiographic data of mice in the Sham group and the D‐gal group. Data are presented as mean ± SD, *n* = 5. *p < 0.05, **p < 0.01, ***p < 0.001.
**Figure S2:** Time‐course validation of D‐gal‐induced cardiomyocyte senescence.
Neonatal mouse cardiomyocytes (NMCMs) were treated with D‐gal for 0, 1, 2, 5, and 10 days, and senescence‐associated parameters were assessed at each time point. (A) Cell viability was measured by CCK‐8 assay. (B) Cell proliferation was evaluated by EDU incorporation assay. (C) Apoptosis rate was determined by flow cytometry. (D) Protein expression levels of senescence‐associated markers p16, p21, and p53 were analyzed by WB. Data are presented as mean ± SD, *n* = 3. *p < 0.05, **p < 0.01, ***p < 0.001.
**Figure S3:** CFTR knockdown exacerbated the D‐gal‐induced reduction in PMCA signaling and calcium dysregulation. (A) The expression of CFTR protein was analyzed by WB. (B) The expression of PMCA protein was analyzed by WB. (C) Levels of Ca2^+^ in NMCMs. (D) Levels of Cl^−^ in NMCMs. Data are presented as mean ± SD, *n* = 3. *p < 0.05, **p < 0.01, ***p < 0.001.
**Figure S4:** Analysis of echocardiographic data for mice in the Sham group, D‐gal group, D‐gal+oe‐NC group, and D‐gal+oe‐USP45 group. Data are presented as mean ± SD, *n* = 5. *p < 0.05, **p < 0.01, ***p < 0.001.

**Data S1:** The ARRIVE guidelines 2.0: author checklist.

## Data Availability

The data that support the findings of this study are available from the corresponding author upon reasonable request.
