# Supplementary material for: A Non‐Channel Function of CFTR: Attenuating Mitochondrial Oxidative Stress and Cardiomyocyte Senescence via Stabilization by USP45
Source: Aging Cell. 2026 Jun 23;25(7):e70610. doi: 10.1111/acel.70610 (PMC13288055; doi:10.1111/acel.70610)
Supplement: Supplementary file 1 — Figure S1: Analysis of echocardiographic data of mice in the Sham group and the D‐gal group. Data are presented as mean ± SD, n = 5. *p < 0.05, **p < 0.01, ***p < 0.001. Figure S2: Time‐course validation of D‐gal‐induced cardiomyocyte senescence. Neonatal mouse cardiomyocytes (NMCMs) were treated with D‐gal for 0, 1, 2, 5, and 10 days, and senescence‐associated parameters were assessed at each time point. (A) Cell viability was measured by CCK‐8 assay. (B) Cell proliferation was evaluated by EDU incorporation assay. (C) Apoptosis rate was determined by flow cytometry. (D) Protein expression levels of senescence‐associated markers p16, p21, and p53 were analyzed by WB. Data are presented as mean ± SD, n = 3. *p < 0.05, **p < 0.01, ***p < 0.001. Figure S3: CFTR knockdown exacerbated the D‐gal‐induced reduction in PMCA signaling and calcium dysregulation. (A) The expression of CFTR protein was analyzed by WB. (B) The expression of PMCA protein was analyzed by WB. (C) Levels of Ca2+ in NMCMs. (D) Levels of Cl− in NMCMs. Data are presented as mean ± SD, n = 3. *p < 0.05, **p < 0.01, ***p < 0.001. Figure S4: Analysis of echocardiographic data for mice in the Sham group, D‐gal group, D‐gal+oe‐NC group, and D‐gal+oe‐USP45 group. Data are presented as mean ± SD, n = 5. *p < 0.05, **p < 0.01, ***p < 0.001. [file ACEL-25-e70610-s001.docx]

**Supplementary Figures**

**
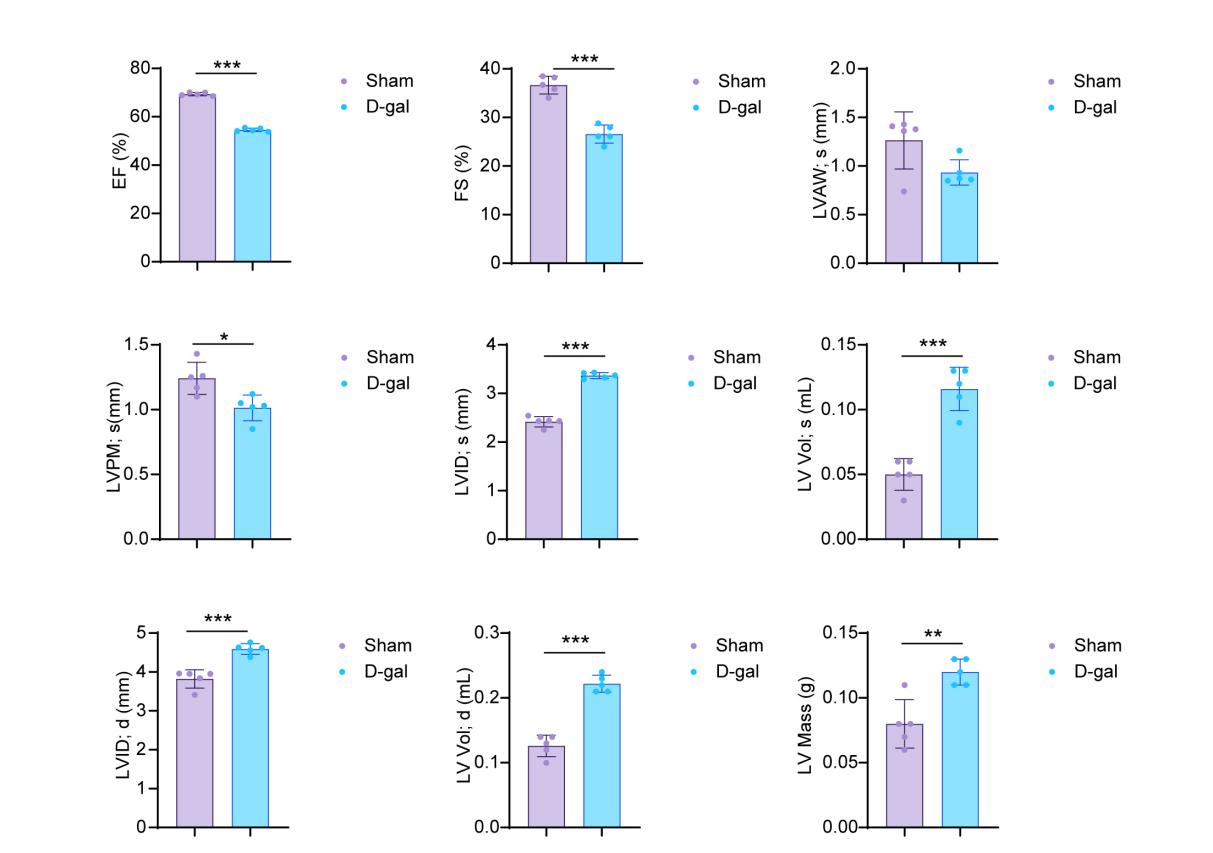
**

**Figure S1.** **Analysis of echocardiographic data of mice in the Sham group and the D-gal group.** Data are presented as mean ± SD, n = 5. **p*<0.05, ***p*<0.01, ****p*<0.001.


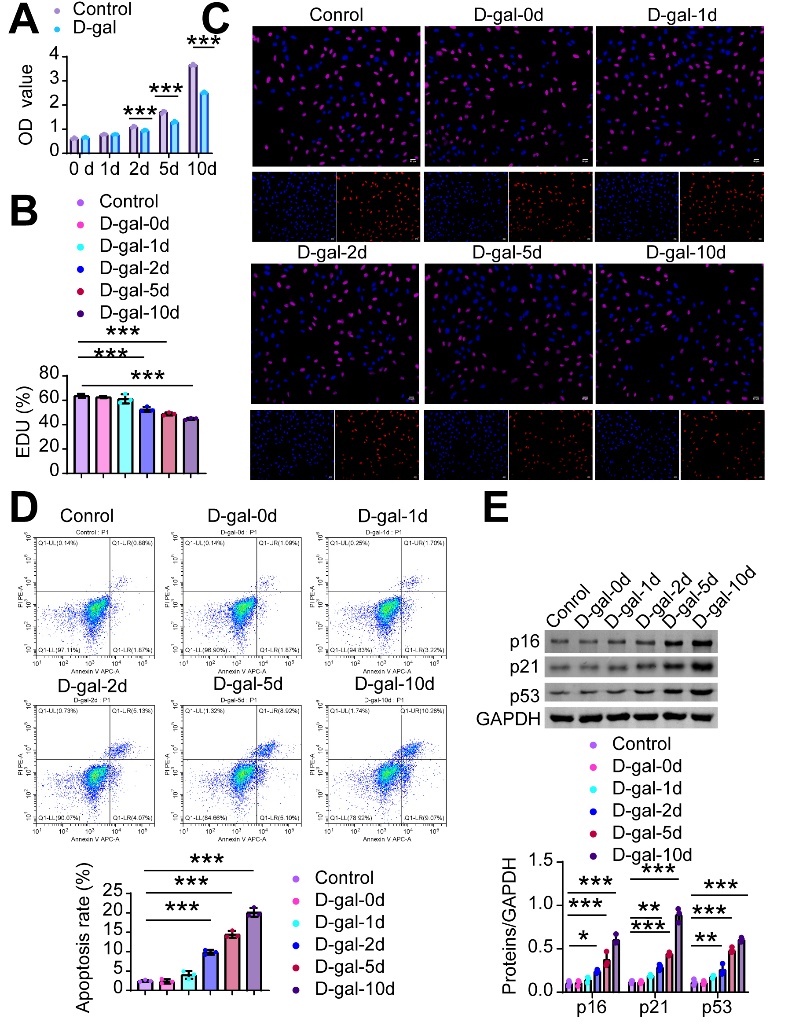


**Figure S2.** **Time-course validation of D-gal-induced cardiomyocyte senescence.
Neonatal mouse cardiomyocytes (NMCMs) were treated with D-gal for 0, 1, 2, 5, and 10 days, and senescence-associated parameters were assessed at each time point.** (A) Cell viability was measured by CCK-8 assay. (B) Cell proliferation was evaluated by EDU incorporation assay. (C) Apoptosis rate was determined by flow cytometry. (D) Protein expression levels of senescence-associated markers p16, p21, and p53 were analyzed by WB. Data are presented as mean ± SD, n = 3. **p*<0.05, ***p*<0.01, ****p*<0.001.


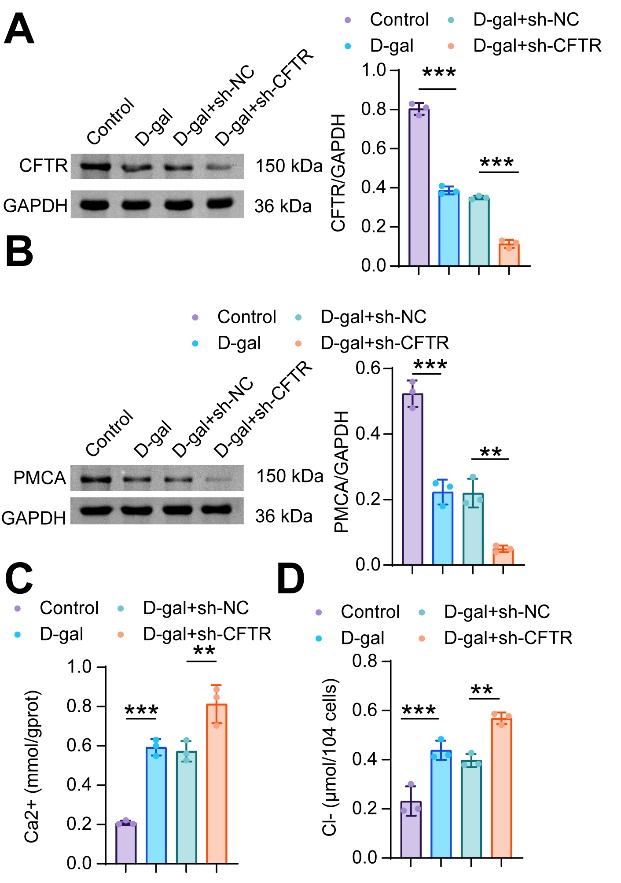


**Figure S3.** **CFTR knockdown exacerbated the D-gal-induced reduction in PMCA signaling and calcium dysregulation.** (A) The expression of CFTR protein was analyzed by WB. (B) The expression of PMCA protein was analyzed by WB. (C) Levels of Ca2^+^ in NMCMs. (D) Levels of Cl^-^ in NMCMs. Data are presented as mean ± SD, n = 3. **p*<0.05, ***p*<0.01, ****p*<0.001.


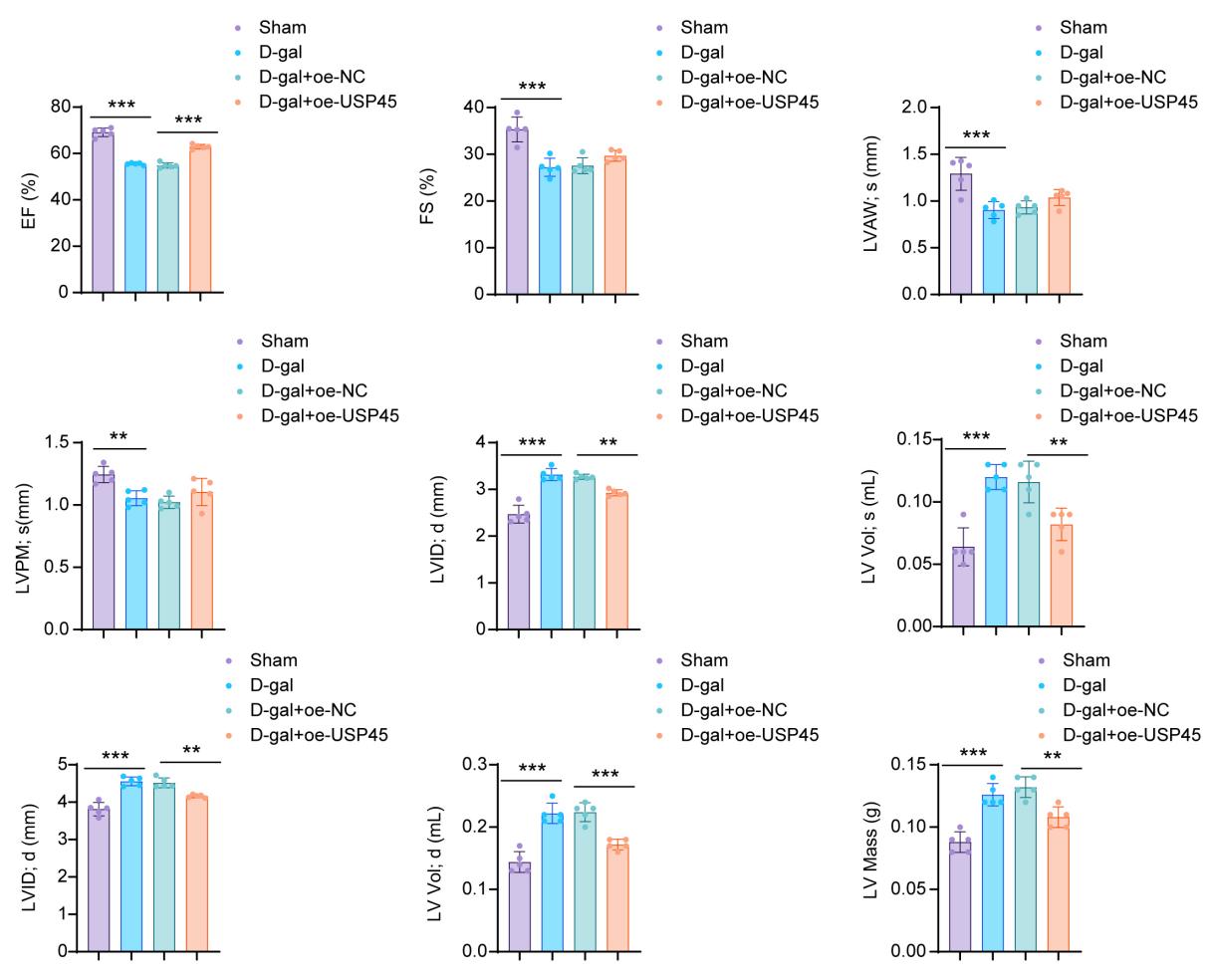


**Figure S4. Analysis of echocardiographic data for mice in the Sham group, D-gal group, D-gal+oe-NC group, and D-gal+oe-USP45 group.** Data are presented as mean ± SD, n = 5. **p*<0.05, ***p*<0.01, ****p*<0.001.
